# New interpretable machine-learning method for single-cell data reveals correlates of clinical response to cancer immunotherapy

**DOI:** 10.1016/j.patter.2021.100372

**Published:** 2021-10-27

**Authors:** Evan Greene, Greg Finak, Leonard A. D'Amico, Nina Bhardwaj, Candice D. Church, Chihiro Morishima, Nirasha Ramchurren, Janis M. Taube, Paul T. Nghiem, Martin A. Cheever, Steven P. Fling, Raphael Gottardo

**Affiliations:** 1 Vaccine and Infectious Disease Division, Fred Hutchinson Cancer Research Center, Seattle, WA, USA; 2 Biostatistics Bioinformatics and Epidemiology Division, Fred Hutchinson Cancer Research Center, Seattle, WA, USA; 3 Clinical Research Division, Fred Hutchinson Cancer Research Center, Seattle, WA, USA; 4 Cancer Immunotherapy Trials Network, Fred Hutchinson Cancer Research Center, Seattle, WA, USA; 5 Division of Dermatology, Department of Medicine University of Washington, Seattle, WA, USA; 6 Bloomberg Kimmel Institute for Cancer Immunotherapy and the Sidney Kimmel Comprehensive Cancer Center, Johns Hopkins University School of Medicine, Baltimore, MD, USA; 7 Tisch Cancer Institute, Icahn School of Medicine at Mount Sinai New York, NY, USA; 8 Centre Hospitalier Universitaire Vaudois et Université de Lausanne, Lausanne, Switzerland

**Keywords:** algorithms, statistics & probability, bioinformatics, immunology, single-cell, cancer

## Abstract

We introduce a new method for single-cell cytometry studies, FAUST, which performs unbiased cell population discovery and annotation. FAUST processes experimental data on a per-sample basis and returns biologically interpretable cell phenotypes, making it well suited for the analysis of complex datasets. We provide simulation studies that compare FAUST with existing methodology, exemplifying its strength. We apply FAUST to data from a Merkel cell carcinoma anti-PD-1 trial and discover pre-treatment effector memory T cell correlates of outcome co-expressing PD-1, HLA-DR, and CD28. Using FAUST, we then validate these correlates in cryopreserved peripheral blood mononuclear cell samples from the same study, as well as an independent CyTOF dataset from a published metastatic melanoma trial. Finally, we show how FAUST's phenotypes can be used to perform cross-study data integration in the presence of diverse staining panels. Together, these results establish FAUST as a powerful new approach for unbiased discovery in single-cell cytometry.

## Highlights

• An interpretable machine-learning method for cytometry data analysis is developed
• Using this, candidate biomarkers of response to therapy are identified and visualized
• The method is used to validate our findings on two additional cytometry datasets
• It is shown how to integrate findings across datasets with heterogeneous marker panels

## The bigger picture

Our article introduces a new method, FAUST, which combines novel algorithms for clustering, cluster matching, variable selection, and feature selection. While these algorithms were developed for application to high-dimensional single-cell data—and our article validates this application area with multiple case studies—they are general purpose and can be applied to any collection of related real-valued matrices one wishes to partition.

Some useful features of these algorithms to the broader data science community include the following: they estimate the number of clusters across a dataset, they can be applied independently to each matrix in the set of matrices one wishes to cluster, they match clusters across matrices on the basis of data-driven annotations, and the annotations are interpretable in relation to the initial measurement variables. We provide an open-source implementation of our method, https://github.com/RGLab/FAUST, targeting data structures optimized for use in cytometry data analysis.

## Teaser

A novel interpretable machine-learning method is developed and applied to discover and visualize candidate biomarkers of response to immunotherapy in single-cell data from a Merkel cell carcinoma clinical trial. We provide demonstrations of how to validate findings on independent cytometry datasets, simulation and benchmark studies comparing our method with existing approaches, and illustrations of how to integrate findings across datasets with diverse staining panels.

## Introduction

Cytometry is used throughout the biological sciences to interrogate the state of an individual's immune system at the single-cell level. Modern instruments can measure approximately 30 (via fluorescence) or 40 (via mass) protein markers per individual cell,^1^^,^^2^ and increasing throughput can quantify millions of cells per sample. In typical clinical trials, multiple biological samples are measured per subject in longitudinal designs. Consequently, a single clinical trial can produce hundreds of high-dimensional samples that together contain measurements on many millions of cells.

To analyze these data, cell subpopulations of interest must be identified within each sample. The manual process of identifying cell subpopulations is called “gating.” In modern high-dimensional panels, gating inevitably introduces bias into cytometry data analysis, since manual gating strategies will only identify cell subpopulations deemed important a priori by the investigator. As the number of possible populations grows exponentially with the number of measured protein markers, manual identification cannot be used to perform unbiased discovery and analysis: there are too many combinations of markers to consider.

Researchers have developed numerous computational methods to address these limitations,^3^, ^4^, ^5^, ^6^, ^7^ helping scientists interrogate the immune system in a variety of clinical settings.^8^^,^^9^ Despite successes, computational gating methods face significant challenges when applied to large experimental datasets. Similar to manual gating, methods often require investigators to bound or pre-specify the number of clusters (i.e., cell subpopulations) expected in a sample,^6^^,^^10^ or to know the relevant clusters in advance.^11^ Such information is often not available. One proposed solution is to partition a dataset into a very large number of clusters in order to capture its main structure.^12^ However, when methods make strong assumptions about the distribution of protein measurements,^13^^,^^14^ the structure captured by over-partitioning can reflect a method's assumptions rather than biological signal.^15^

Another challenge for many methods is that biologically equivalent clusters are given arbitrary numeric labels when samples are analyzed independently. In such cases, methods must provide a way to match biologically comparable clusters across samples. One matching approach is to quantify cluster similarities across samples with a user-specified metric.^16^^,^^17^ As the dimensionality of the data increases, choosing an appropriate metric becomes more difficult due to sparsity.^12^ An alternative approach is to concatenate samples together and then cluster the combined data.^4^^,^^18^^,^^19^ However, this approach can mask biological signal in the presence of batch effects or large sample-to-sample variation in protein expression. It also introduces the risk that a method will fail to identify small but biologically interesting clusters, since computational considerations can lead authors to recommend subsampling cells from each sample before combining the samples for analysis.^5^

**Figure 1:**
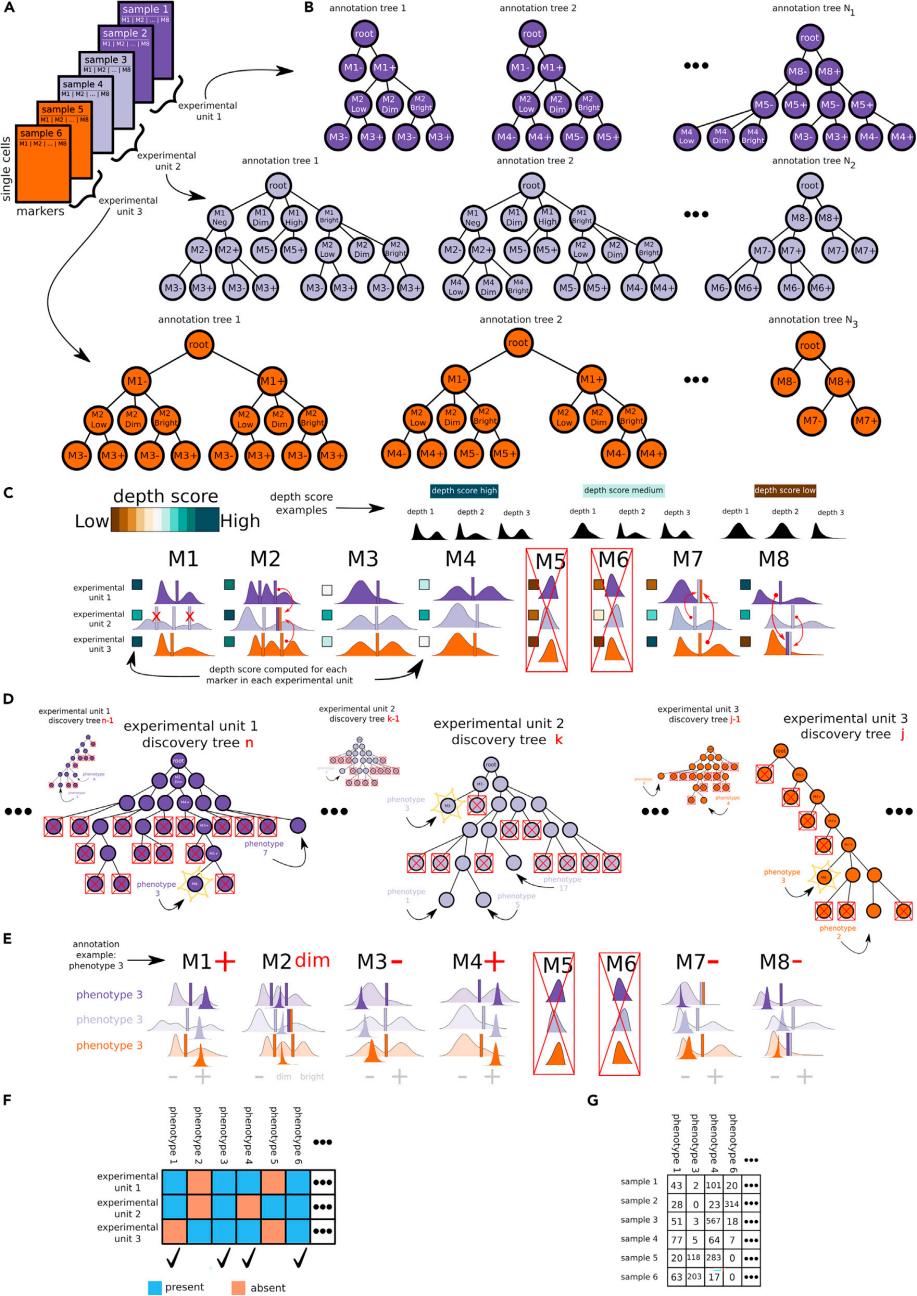
FAUST overview (A) Samples with markers M1 to M8 are grouped into experimental units (EUs) that are concatenated for analysis. (B) FAUST exhaustively explores the space of “reasonable” 3-marker gating strategies for each EU to compute an annotation forest. (C) Using this, FAUST scores each marker in each EU and selects consistently high-scoring markers for continued analysis (M5, M6 are removed here). Thresholds are standardized across EUs for selected markers: if a selected marker has EUs in which the number of estimated thresholds does not agree with the standard, thresholds are either removed (M1, EU2) or imputed (M2, EU2; M7, EU1; M8, EU3) using information from EUs adhering to the standard (denoted by the red arrows). (D) Discovery forests are then grown for each EU. Each leaf of each tree corresponds to a phenotype. All phenotypes are scored across forests, high-scoring phenotypes are selected (leaf nodes without a red ×; starred nodes subsequently survive down-selection in F), and low-scoring phenotypes are discarded (leaf nodes with red ×). (E) Selected phenotypes are annotated using the standardized thresholds from (C). (F) Phenotypes are down-selected based on frequency of occurrence across EUs. (G) A per-sample count matrix is derived for down-selected phenotypes.

To address these challenges, we have created an interpretable machine-learning method that discovers and annotates cell populations across cytometry experiments, named Full Annotation Using Shaped-constrained Trees (FAUST, Figure 1). FAUST solves these issues by combining novel algorithms for variable selection, clustering, cluster matching, and feature selection. The key assumption underpinning FAUST is that biologically relevant clusters of cells separate into homogeneous modal groups along the markers measured in a panel. Building off this assumption, the FAUST method systematically uses one-dimensional non-parametric tools to discover and annotate cellular phenotypes.

FAUST is interpretable in that its final product is an annotated matrix of counts for an experiment, with rows corresponding to samples and columns to annotated selected features. FAUST features are defined by their annotations: for a given marker panel, they are the set of cells in each sample that fall within the region of marker co-expression asserted by the annotation. These features are interpretable, since the co-expression patterns in the annotation can be used directly to map features onto known immunological populations, such as B cells and T cells. Examples of this mapping process are given throughout this paper (e.g., the last four subsections of the results). Binding feature definitions to their phenotypic annotations within each sample distinguishes FAUST both from clustering methods that return numeric cluster labels and biologically interpretable analysis approaches that rely on pre-specifying reference subsets across an experiment to which biological annotations are subsequently assigned^20^ or which have biological interpretation due to their pre-specification strategy.^21^

At least one marker in a dataset must exhibit multimodality to use FAUST: we have observed this condition usually holds in high-throughput samples stained with high-dimensional panels. When the assumption is met, FAUST can discover clusters with phenotypic labels sample-by-sample, match clusters across samples using these phenotypes, and gate out a subset of phenotypes that are consistently discovered across the experiment. Unlike manual gating, FAUST is unbiased because it estimates the number of phenotypes in a data-driven fashion. In contrast to many existing methods, FAUST's ability to discover subsets on a per-sample basis and then match subsets by their label allows FAUST to analyze large cytometry experiments without concatenating the entire dataset.

FAUST facilitates biological discovery and validation: any phenotype discovered by FAUST found to be significantly associated with an outcome of interest in downstream modeling can be targeted in independent datasets for validation. We demonstrate this workflow by applying FAUST to data from a Merkel cell carcinoma (MCC) anti-PD-1 trial, and identifying several effector memory T cell phenotypes whose presence in blood at baseline (pre-treatment) are significantly associated with subjects' positive response to therapy. We then use FAUST to validate these associations by targeting the comparable effector memory T cell phenotypes in baseline anti-PD-1 data from a previously published metastatic melanoma trial, as well as cryopreserved peripheral blood mononuclear cells (PBMCs) from the same study, and demonstrate that the same positive associations hold in both datasets.

In addition to targeted hypothesis testing, FAUST enables a multivariate approach to hypothesis testing we call “Phenotypic and Functional Differential Abundance” (PFDA), inspired by techniques used in gene set enrichment analysis.^22^ PFDA fits a multivariate model to all FAUST cell subpopulations whose phenotypes are consistent with a pre-specified marker combination, then uses the estimated model coefficients to conduct tests of differential abundance. We contrast PFDA with targeted hypothesis testing by applying FAUST to myeloid and T cell datasets from three independent cancer immunotherapy trials, then use both approaches to test hypotheses concerning simple phenotypes. We demonstrate that both approaches offer useful ways to perform cross-study analysis and also show that PFDA can detect signals missed by the targeted approach if the targeted phenotype is mis-specified.

In total, we apply FAUST to seven cytometry datasets generated from five independent studies. We demonstrate how FAUST can be used to discover candidate biomarkers associated with treatment outcome, validate these associations on independent data, and perform cross-study analyses in the presence of heterogeneous marker panels.

## Results

### The FAUST method

Intuitively, FAUST consists of two main phases. The first phase consists of deriving a standardized set of thresholds for informative markers in the cytometry experiment (Figures 1A–1C). FAUST uses the standardized thresholds to define biologically interpretable features with phenotypic labels. These labels are later used to match features across experimental samples. FAUST can estimate thresholds on a per-sample basis, and uses information about the modal structure of marginal and conditional densities in each sample to make its estimate. The reason standardization is required is because FAUST can operate sample by sample: if a cellular population is either absent from or only present in a minority of experimental samples, the modal structure in that subset will be different from the majority of the experiment. This structural difference must be resolved in order to match phenotypic features across the experimental samples, since FAUST ultimately defines features relative to the set of thresholds for a given sample. This requirement motivates the first phase of FAUST.

The second phase of FAUST consists of discovering and down-selecting phenotypes in terms of the informative markers (Figures 1D–1G). As with the thresholds, phenotypes can be discovered on a sample-by-sample basis. This is done by growing numerous trees whose leaves correspond to candidate phenotypes and then selecting a subset of leaves across trees that jointly partition the sample. The intuition behind this procedure is that since the trees are generated using one-dimensional density estimates, we expect that any given tree will mostly have leaves arrived at from suboptimal partitioning strategies. This is because the strategies are not directly capturing the higher-dimensional structure of the data at any step. However, we further intuit that by exploring complex strategies enabled by the high-dimensional nature of the data, in some trees a few leaves will be produced by relatively optimal strategies that identify homogeneous collections of cells. Thus, by growing a very large number of trees and selecting leaves across them, the procedure attempts to produce a partition of each sample consisting solely of high-quality leaves. Once partitioned, FAUST identifies the phenotype of each selected leaf by determining where its marginal expression distributions fall relative to its sample's standardized thresholds. This generates a list of phenotypes discovered in each sample. FAUST then down-selects to the set of phenotypes that frequently occurs across experimental samples based on the hypothesis that the phenotypes in this set represent actual structure (rather than being methodological artifacts), since they are discovered independently across samples. FAUST concludes by constructing a matrix of counts for these down-selected phenotypes. The remainder of this section provides additional details about the components of each phase.

To apply FAUST to a cytometry dataset, an analyst first sets several parameters describing the experimental design to the method. These parameters include specifying the starting population of cells to analyze in each sample (if pre-gating of the data is available) and specifying which markers to use in the analysis. The key parameter to set is the experimental unit, which determines whether any groups of samples should be concatenated prior to discovery and annotation of phenotypes (Figure 1A). By default, the experimental unit is set to individual samples. However, in longitudinal experiments where samples from a single subject are exposed to multiple stimulations at multiple time points, concatenating samples from a single subject across stimulation conditions may be desirable (“FAUST method: tuning parameters” discusses additional tuning parameters one can set in the FAUST method).

Once parameters have been set, FAUST grows an annotation forest for each experimental unit (Figure 1B). FAUST defines the annotation forest as the exhaustive collection of all “reasonable” 3-marker gating strategies in an experimental unit. FAUST's operative definition of “reasonable” is those combinations of markers for which the null hypothesis of unimodality according to the dip test^23^ is rejected (“FAUST method: growing the annotation forest” gives a complete description). Once grown, FAUST computes a depth score for each marker that quantifies how consistently a marker separates into subpopulations in the annotation forest (see “FAUST method: depth score computation”). FAUST uses the depth score to select a subset of markers in the panel that are consistently high-scoring across experimental units, then estimates a standard number of sample-specific annotation thresholds for each selected marker (Figure 1C). FAUST standardizes the number of thresholds for each selected marker in order to create a common system of annotation for each experimental unit. This annotation system is ultimately used by FAUST to define its clusters and match them across experimental units.

After marker selection and threshold standardization, FAUST grows multiple discovery forests for each experimental unit (Figure 1D). Unlike the trees in the annotation forest, these trees are not depth constrained. Consequently, each tree in the forest is a proposed clustering of the experimental unit, and each leaf within a tree corresponds to a unique phenotype. FAUST scores all leaves of all trees in the discovery forests, and selects a subset of high-scoring leaves across multiple trees (see “FAUST method: phenotype discovery and cluster annotation”). The subset of selected leaves jointly partition the experimental unit and are annotated using the experimental unit's standardized thresholds (Figure 1E). As mentioned previously, these phenotypic annotations are then used to match clusters across experimental units. Phenotypes are further down-selected according to how frequently FAUST discovers them across the experimental units (Figure 1F and “FAUST method: tuning parameters” provide details about the down-selection procedure). FAUST concludes by constructing a sample-by-phenotype count matrix for the down-selected phenotypes (Figure 1G). This count matrix is the primary output of the FAUST analysis and can be used in downstream modeling to test discovered phenotypes for association with outcomes of interest.

Because of down-selection, the phenotypes in the count matrix do not account for every cell in the experiment: they comprise a subset of consistently detected cell subsets discovered and selected by FAUST. Filtering to return consistently detected features is a desirable aspect of the method, since it increases the power to detect significant correlates in downstream modeling. That said, FAUST does produce a phenotypic label for every cell in every sample according to the cell's position relative to the standardized annotation thresholds. We developed a novel visualization of the cytometry data based off these annotations, which we illustrate in “FAUST identifies and visualizes baseline T cells in blood associated with outcome in CITN-09, a Merkel cell carcinoma anti-PD-1 trial.” First, however, here we present data from simulation studies showing that the consistently detected features returned by FAUST correspond well to components in high-dimensional mixture models.

### FAUST resolves high-dimensional structure in simulation studies

We performed simulation studies in two settings: a “control” setting in which we generated samples from multivariate Gaussian mixtures, and a “stimulation” setting in which we transformed the data so their margins were no longer Gaussian. We viewed the Gaussian setting as a “control” because implicit Gaussian assumptions are encountered in widely used methods.^24^^,^^25^ We expected most methods to perform well in this setting. Our goal was to assess each method's performance at the following tasks: recovering the true number of clusters; correctly assigning abundances to each cluster; and discovering the specific cluster that predicts a simulated outcome.

We first generated 10 samples from multivariate Gaussian mixtures, identifying mixture components as clusters (a 2-component mixture has been discussed previously).^26^ We varied the number of components from 5 to 115. We applied FAUST, DEPECHE,^27^ flowMeans,^10^^,^^28^ FlowSOM,^6^^,^^29^ *k*-means, PARC,^30^ and PhenoGraph^31^^,^^32^ to the simulated datasets (supplemental experimental procedures A.7 shows an example of the simulated data). We provided *k*-means with the true number of clusters in each simulation iteration, a required parameter setting. Due to its computational efficiency, we applied FlowSOM in different ways, both letting it estimate the number of clusters and providing that information (deemed “oracle” settings, see “simulation study: estimating the number of clusters and partition scoring”).

**Figure 2:**
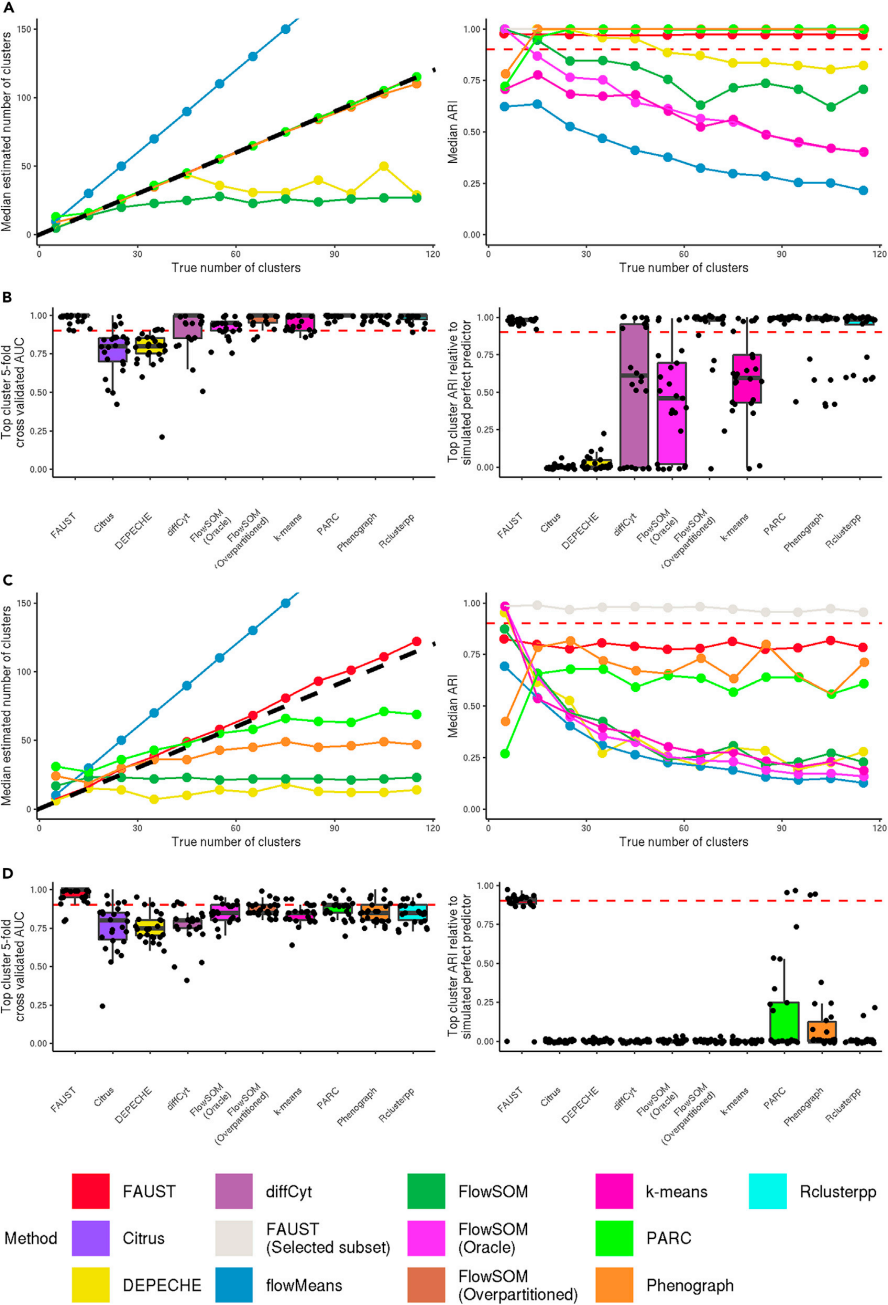
Simulation studies (A) Left: median estimated number of clusters by method, 5 simulation iterations for each truth value, multivariate Gaussian setting. Right: median adjusted Rand index (ARI) by method, 5 simulation iterations for each truth value, multivariate Gaussian setting. (B) Left: cross-validated AUC of the top cluster for each of 25 iterations, multivariate Gaussian setting, points jittered by a maximum of 0.0125. Right: median ARI between a method's top cluster and the simulated true predictor, 10 iterations per expected fold change, multivariate Gaussian setting. (C and D) All panels report results from applied methods to simulated datasets transformed by Γ(1+|x/4|). (C) Left: same as (A, left). Right: same as (A, right). (D) Left: same as (B, left). Right: same as (B, right). Horizontal dashed red line at 0.90.

We repeated this simulation five times per setting and recorded the median number of clusters (for methods that estimated it). For FAUST, we identified the number of down-selected phenotypes as the number of clusters. We also recorded the median adjusted Rand index (ARI) relative to the simulated truth for each method, both on the entire dataset and the subset of observations consisting of FAUST's down-selected phenotypes. In this scenario, FAUST, PARC, and PhenoGraph were consistently able to estimate close to the true number of clusters; these same methods had median ARI above 0.9 across simulation settings (Figure 2A).

To test FAUST as a discovery tool, we simulated a dataset composed of 20 samples drawn from multivariate Gaussian mixture models with 125 components. The weight vector of the mixture was fixed for 10 “non-responder” samples. For the other 10 “responder” samples, the weight of a single targeted component was doubled by proportionally decreasing weight from the other 124 components. This simulated a perfect predictor of responder status: samples with the elevated component were responders; those without, non-responders.

We applied FAUST to 25 datasets simulated under these conditions. Since we simulated a responder status, we also applied citrus,^5^ Rclusterpp,^33^ and diffCyt,^19^^,^^34^ as well as the previous methods (omitting flowMeans due to computational constraints). For each method, the top cluster associated with responder status was identified (see “simulation study: predicting simulated responder status;” note that for citrus and diffCyt we attempted to use the modeling built into those methods to identify the “top cluster”). We then used a logistic model to predict responder status using the frequency of each method's top cluster in order to normalize across different approaches to predictive modeling. We assessed performance using 5-fold cross-validated area under the ROC curve (5cvAUC).^35^ We also computed the ARI of each method's top cluster relative to the simulated perfect predictor. We found that the top cluster from multiple methods had median 5cvAUC above 0.9 and median ARI above 0.9 across the 25 iterations (Figure 2B).

Next, we repeated the simulation studies with one change: each column of each simulated dataset was transformed by the map Γ(1+|x/4|) in order to test the performance on non-Gaussian data. This transformation was selected to produce datasets with marker distributions that visually resembled those in CyTOF datasets with randomization applied (supplemental experimental procedures A.7). Among the tested methods, FAUST best estimated the number of clusters in the dataset and had median ARI above 0.9 on the annotated subset (Figure 2C and supplemental experimental procedures A.10 together demonstrate that non-FAUST methods have similar median ARI on and off the selected subset). In addition, FAUST's top cluster had median ARI above 0.9 across the 25 iterations (Figure 2D).

### FAUST discovers predictive phenotypes in benchmark dataset

We applied FAUST to the FlowCAP-IV challenge dataset to evaluate its performance relative to existing methods on a known benchmark.^36^ The FlowCAP-IV dataset consists of 766 paired PBMC samples measured by flow cytometry from 383 HIV patients, split into a training set of 382 samples from 191 patients, and a test set of 384 samples from 192 patients. Each patient provided two samples: an unstimulated control sample and a sample stimulated by HIV-Gag peptides. The FlowCAP-IV challenge tasked methods with discovering cellular subsets on the training set that could predict time to progression to AIDS on the test set.

FloReMi,^37^ the best-performing approach from the original publication, combined data pre-processing, feature extraction, feature selection, and prediction. Method performance was summarized using a Cox proportional hazards model, with predicted survival times on the test set as a predictor variable. FloReMi reported a $-{\text{log}}_{10}\left(\text{p}\;\text{value}\right)$ of 2.699 and a concordance index of 0.672 on the test set.

We observed that FloReMi could be modified to use FAUST for feature extraction and feature selection. This was only possible because of FAUST's phenotypic annotations: we were able to apply FAUST to the training set, discover and annotate phenotypes, and then subsequently use FAUST to derive counts on the test set specifically for the phenotypes that had been independently selected on the training set. When we used FAUST to define predictive features in this way (see “flowCAP-IV analysis”), and combined these features with FloReMi's pre-processing and predictive framework, we observed a $-{\text{log}}_{10}\left(\text{p}\;\text{value}\right)$ of 4.243 and a concordance index of 0.720 on the test set.

### FAUST identifies and visualizes baseline T cells in blood associated with outcome in CITN-09, a Merkel cell carcinoma anti-PD-1 trial

We used FAUST to perform phenotype discovery in cytometry data generated from fresh, whole blood isolated from patients with Merkel cell carcinoma (MCC) receiving pembrolizumab on the Cancer Immunotherapy Trials Network (CITN) phase 2 clinical trial CITN-09,^38^ with the goal of identifying baseline correlates of response to treatment (NCT02267603, see supplemental experimental procedures A.1). We analyzed 78 longitudinal samples stained with immunophenotyping panels to identify T cell subsets within whole blood (see “CITN-09 T cell panel analysis”). We used binomial generalized linear mixed models (GLMMs)^39^ to test each FAUST phenotype for differential abundance at the baseline time point (prior to receiving anti-PD-1 therapy) between responders and non-responders in 27 subjects (Equation 4.2). Responders were defined as subjects who exhibited either a complete response (CR) or partial response (PR) (as per RECIST 1.1)^40^ and non-responders as subjects exhibiting progressive disease (PD) or stable disease (SD).

**Figure 3:**
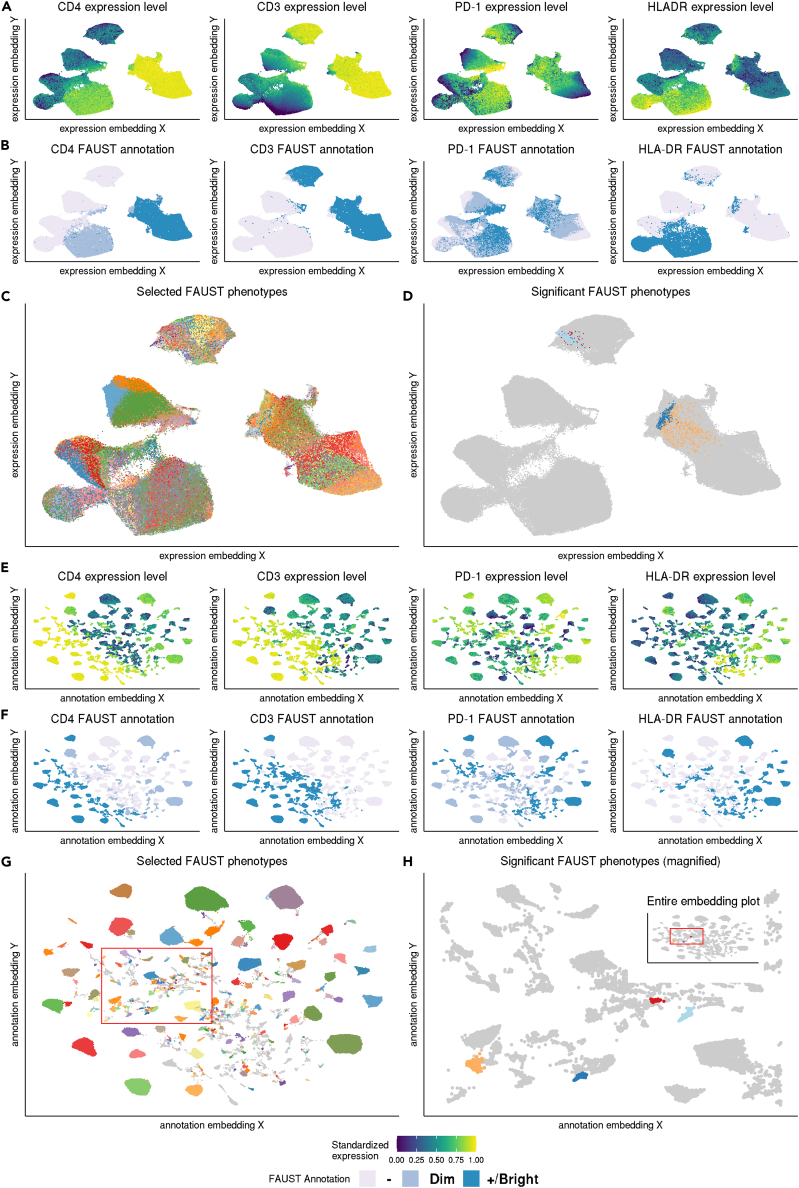
FAUST annotations enable novel embeddings that reflect expression differences not captured by direct dimensionality reduction UMAP embedding of the observed expression matrix colored by: (A) the expression for the stated marker winsorized at the 1st and 99th percentile, and scaled to the unit interval; (B) the associated per-cell FAUST annotation; (C) all selected FAUST phenotypes; (D) significant FAUST phenotypes. UMAP embedding of the annotation transformed expression matrix with (E) colored as (A), (F) colored as (B), (G) colored as (C), and (H) colored as (D). The red bounding box in (G) and (H) contains the four significant correlates discovered in the FAUST analysis. The inset in (H) is the entire embedding plot; the main component is zoomed into the bounding box to show the relative placement of the four correlates on the annotation embedding.

At a Bonferroni-adjusted 10% level, four FAUST phenotypes were significantly associated with response to therapy (Figure 3D). Two had a CD28^+^ HLA-DR^+^ CD8^+^ effector memory phenotype, and were annotated either PD-1^dim^ or PD-1^bright^, respectively. The other two were CD4 bright: one had an HLA-DR^−^ CD28^+^ PD-1^dim^ phenotype and the other an HLA-DR^+^ CD28^+^ PD-1^dim^ phenotype (data for both CD4 phenotypes is shown in supplemental experimental procedures A.6). The observed CD28^+^ phenotypes agree with published findings highlighting the importance of CD28 expression in CD8^+^ T cells in anti-PD1 immunotherapy.^41^^,^^42^ Effect sizes with 95% confidence intervals for the correlates are reported in supplemental experimental procedures A.14. FAUST annotated all four correlates as CD45RA^−^ and CCR7^−^, indicating that they represented effector memory T cells.

To visualize these data, we applied UMAP^43^ to the primary flow cytometry data with “qualitative” parameter settings^44^ (Figures 3A–3D). The UMAP “islands” in the resulting embedding contained relatively homogeneous expression of several measured protein markers such as CD3 and CD4. Those same islands contained noticeable variation in expression of other markers such as PD-1 and HLA-DR (Figure 3A). The variability of marker expression observed in UMAP “islands” was also observed in the single-cell FAUST annotations for individual markers (Figure 3B). Because of this variation, selected FAUST phenotypes exhibited considerable within-island overlap when displayed on these UMAP embeddings (Figure 3C). The four significant FAUST phenotypes were not distinguishable solely on the basis of the UMAP embedding of the primary expression data (Figure 3D).

To improve the visualization of FAUST results, we developed a novel approach to embedding cytometry data, which we call generating an “annotation embedding” (see “FAUST algorithm: annotation embedding”). In brief, this approach works by using each observed FAUST annotation (both selected and filtered phenotypes) as a “landmark.” All expression data corresponding to a distinct phenotype is shifted and scaled marker by marker so that the marker data is centered at and standardized around the appropriate “landmark.” The transformed dataset is then embedded using the dimensionality reduction algorithm of choice; here we continue to use the UMAP algorithm.

When we applied this approach to the MCC cytometry data, we observed that within-island marker expression in the annotation embedding appeared more homogeneous across the selected markers relative to the embedding generated from the primary expression data (Figure 3E). As a result, FAUST single-cell annotations mapped neatly to each “island” (Figure 3F). This produced visualizations of the selected FAUST phenotypes that did not significantly overlap within islands (Figure 3G). Consequently the four significant FAUST phenotypes were clearly displayed in the annotation embedding (Figure 3H).

### CD8^+^ T cells from virus-positive subjects correlate with in-tumor measurements; their association with outcome validates on independent data

**Figure 4:**
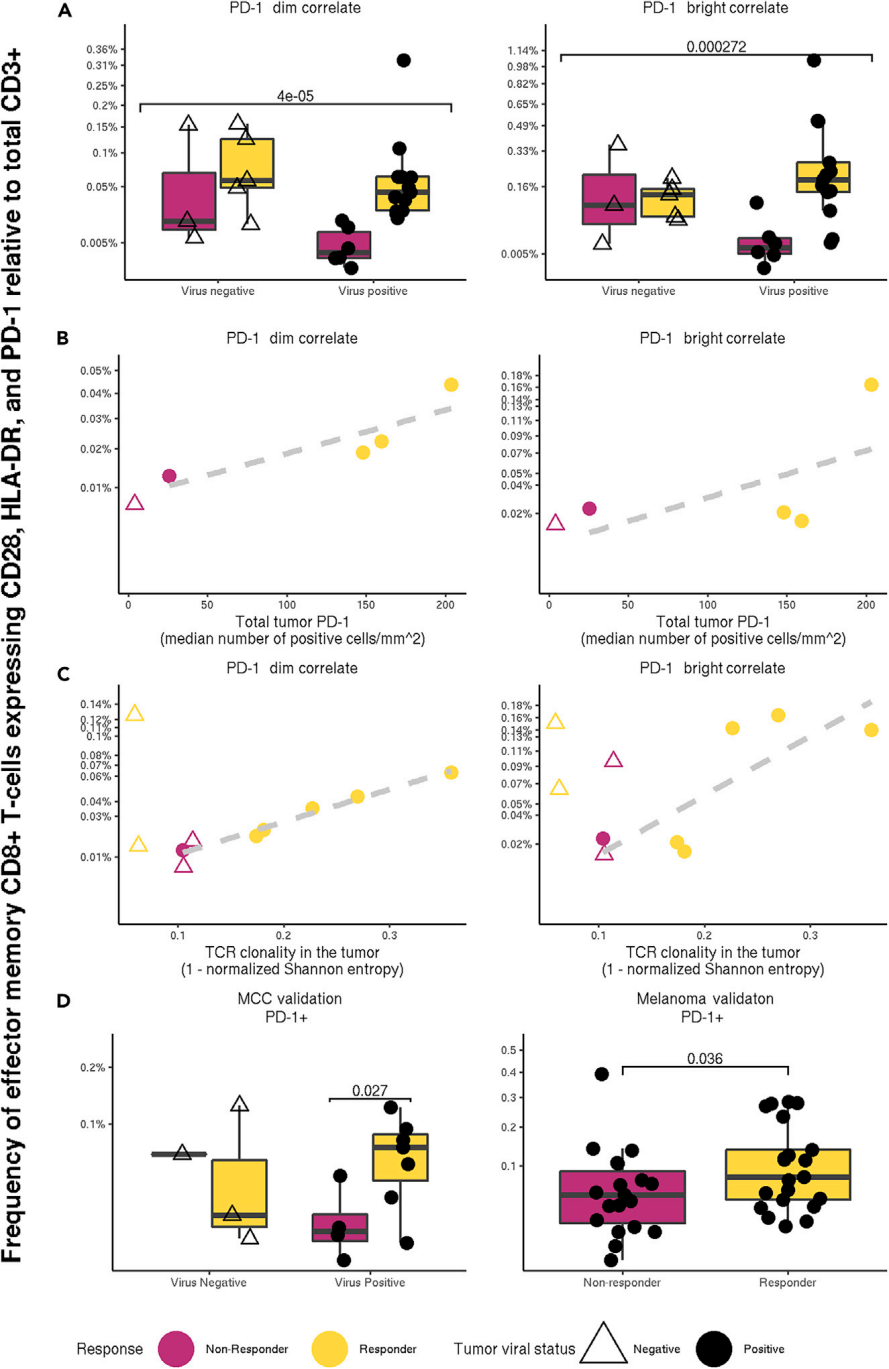
FAUST CD8^+^ phenotypes are associated with positive response to anti-PD-1 therapy in virus-positive subjects (A–C) (A) The two CD8 FAUST phenotypes significantly associated with positive treatment outcome, stratified by viral status. Observed p values contrasting all responders (n = 18) against all non-responders (n = 9) are reported in the figure. Frequencies of the CD8^+^ phenotypes relative to total CD3^+^ cells versus (B) total PD-1 expression measured by IHC from tumor biopsies as described in Giraldo et al.^46^ (C) Productive clonality (1 − normalized entropy) from tumor samples as described in Miler et al.^47^ A suggestive trend is observed in both (B) and (C) among virus-positive subjects, although strong conclusions are not warranted due to the small sample size. (D) Targeted frequencies relative to total CD3^+^ cells in the cryopreserved PBMC samples (MCC validation) with observed p value contrasting responders against non-responders in virus-positive subjects, and the CyTOF melanoma dataset with observed p value.

MCC is a virus-associated malignancy: Merkel cell polyomavirus occurs in many MCC tumors.^38^ Reports that CD8 T cells co-expressing HLA-DR and CD28 can exhibit anti-viral properties,^45^ as well as reports of CD28-dependent rescue of exhausted CD8 T cells by anti-PD1 therapies in mice,^42^ led us to investigate the association between the abundance of the therapeutic-response-associated phenotypes discovered by FAUST and tumor viral status of each subject, as we hypothesized that these cells may represent virus-specific subpopulations. We adapted the differential abundance GLMM to test for an interaction between response to therapy and tumor viral status in the four significant FAUST phenotypes and found a statistically significant interaction in the CD8 phenotypes (Figure 4A). This suggested that the CD8^+^ T cells may be particularly relevant to anti-tumor response in subjects with virus-positive tumors.

To further investigate the relevance of these CD8^+^ T cells, we examined published data on PD-1 immunohistochemistry (IHC) staining in tumor biopsies from the same patients.^46^ Importantly, the in-tumor PD-1 measurement is a known outcome correlate in MCC.^46^ Limited overlap between the assays resulted in only five subjects for whom both flow cytometry and tumor biopsy anti-PD-1 IHC staining were available, and only four of these were virus positive. Nonetheless, the frequencies of the CD8^+^ T cells were strongly correlated (PD-1^dim^ ρ=0.806; PD-1^bright^ ρ=0.579) with the PD-1 total IHC measurements within the four virus-positive subjects (Figure 4B). We also examined published T cell receptor clonality data generated from patient tumor samples.^47^ Ten subjects passing clonality quality control (QC) were common to the two datasets, six of which were virus positive. Frequencies of the CD8^+^ FAUST populations within these six subjects were strongly correlated (PD-1^dim^ ρ=0.988; PD-1^bright^ ρ=0.790) with the measurement of productive clonality (Figure 4C). We note that the small sample size limits the strength of inferences that can be drawn from these observed correlations.

To validate our findings in the MCC study, we tested the hypotheses that increased abundance of the T cell phenotypes are associated with positive response to pembrolizumab treatment in two different datasets. The first dataset consisted of 15 cryopreserved PBMC patient samples from the MCC study. The second dataset was a published metastatic melanoma CyTOF dataset we downloaded from FlowRepository.^48^ A full description of the latter dataset is given by Subrahmanyam et al.;^49^ here, we restricted our analysis to unstimulated baseline PBMC samples.

In both datasets, we applied FAUST to generate data-driven annotation thresholds for the 10 selected markers used to define phenotypes in the initial MCC analysis. We then used these thresholds to extract counts of the pre-specified T cell correlates in both datasets. Since FAUST defined only one threshold for PD-1 in both of the validation datasets, we targeted PD-1^+^ cells in place of PD-1^dim/bright^ cells. To validate the associations, we then tested the targeted phenotypes for differential abundance between responders and non-responders in samples from subjects that went on to receive pembrolizumab. In the cryopreserved PBMC data, we detected significantly increased abundance among virus-positive subjects in the CD8^+^ T cells; in the melanoma dataset, we detected significantly increased abundance among responding subjects (Figure 4D). We note that in the CyTOF melanoma dataset, responders were defined as patients that exhibited progression-free survival for at least 180 days after therapy.^49^ These analyses validated the associations detected by FAUST in the MCC trial and highlight a powerful feature of FAUST: performing targeted validation across studies and technologies.

### FAUST phenotypes capture underlying biological and technical signals in longitudinal studies

Assessing the sensibility of a method's partitioning of a biological dataset is challenging in the absence of a source of ground truth such as human labels. FAUST's annotations can ameliorate this issue by allowing an investigator to check phenotypes discovered by the method for expected biological or technical effects.

**Figure 5:**
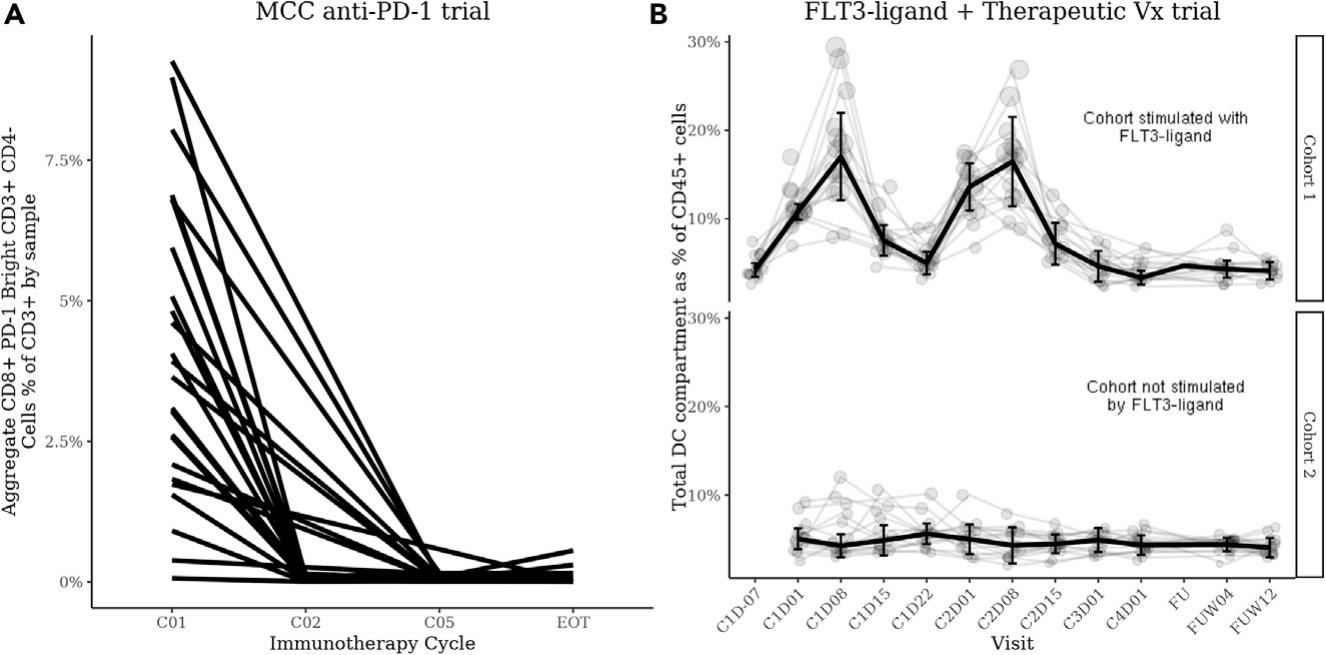
Longitudinal profiles of aggregated FAUST cell populations in a pembrolizumab therapy trial and an FLT3-L + CDX-1401 trial are consistent with underlying technical and biological signals (A) The aggregate frequency of all phenotypes discovered by FAUST containing the subphenotype CD8^+^ PD-1^bright^ CD3^+^ CD4^−^ across all time points. Aggregation occurs within subject by time point. (B) The longitudinal profiles of all cell subpopulations with phenotypes consistent with the DC compartment: CD19^−^, CD3^−^, CD56^−^, HLA-DR^+^, CD14^−^, CD16^−^, and CD11C^+/−^. Light-colored lines show individual subjects. The dark line shows the median across subjects over time. Error bars show the 95% confidence intervals of median estimate at each time point. Cohort 1, n = 16 subjects; cohort 2, n = 16 subjects.

To demonstrate this, we examined the longitudinal profiles of specific phenotypes in the MCC anti-PD-1 trial for which we expected longitudinal changes in the abundance due to known technical effects. Specifically, we examined all selected FAUST phenotypes whose annotations included CD3^+^, CD8^+^, CD4^−^, and PD-1^−/bright^. The temporal abundance of these cells reveals that these cells are not detectable in most samples after subjects have received pembrolizumab therapy (Figure 5A), presumably from pembrolizumab blocking the detecting antibody. The observed decay post treatment is consistent with the manual gating of CD3^+^ CD8^+^ PD-1^+^ cells in this study (supplemental experimental procedures A.5).

We also analyzed flow cytometry data from a second CITN trial: CITN-07 (NCT02129075, see supplemental experimental procedures A.1 for trial data), a randomized phase II trial studying immune responses against a DEC-205/NY-ESO-1 fusion protein (CDX-1401) and a neoantigen-based melanoma vaccine plus poly-ICLC when delivered with or without recombinant FLT3 ligand (CDX-301) in treating patients with stage IIB to stage IV melanoma. The cytometry data consisted of fresh whole blood stained for myeloid cell phenotyping (see “CITN-07 phenotyping panel analysis”).

In the FLT3-ligand + therapeutic Vx trial we expected to observe expansion of dendritic cells in response to FLT3-L stimulation.^50^ After applying FAUST to this dataset, we examined the longitudinal profile of phenotypes whose annotations were consistent with dendritic cells (Figure 5B). This examination revealed dynamic expansion and contraction of the total DC compartment in the FLT3-L-stimulated cohort but not in the unstimulated-by-FLT3-L-pre-treatment cohort. The expansion peaked at day 8 after FLT3-L simulation in cycles 1 and 2. This dynamic is consistent with observations from manual gating of the DC population,^51^ the expected biological effect of FLT3-L,^50^ and the timing of FLT3 administration.

These results demonstrate that FAUST's phenotypes provide a unique way to validate cluster quality in the absence of human labels: FAUST phenotypes can be checked for consistency with technical signals or biological responses that are expected from the experimental design. The longitudinal behavior of PD-1^bright^ T cell populations in the MCC anti-PD-1 trial and the dendritic cells in the FLT3-ligand + CDX-1401 trial are consistent with manual gating of cytometry data and serve as an internal validation of the methodology.

### FAUST enables targeted hypothesis testing for pre-specified phenotypes

FAUST provides investigators with two ways to test pre-specified hypotheses in cytometry studies. We call these procedures “the targeted approach” and PFDA. The targeted approach uses FAUST's standardized thresholds to extract counts for phenotypes matching a pre-specified marker combination. This is the method used on the test set in the benchmark analysis of the section “FAUST discovers predictive phenotypes in benchmark dataset” as well as in the validation analyses of “CD8+ T cells from virus-positive subjects correlate with in-tumor measurements; their association with outcome validates on independent data.” To apply it, an investigator must have a priori knowledge of the exact phenotype to evaluate. In contrast to this, PFDA fits a multivariate model to all FAUST phenotypes that contain a specific subphenotype within their annotations, providing protection against mis-specification. Using PFDA, tests of differential abundance between classes are then performed using linear combinations of the estimated model coefficients. Similar to gene set enrichment analysis methods,^22^ PFDA accounts for the correlation between the different phenotypes discovered by FAUST.

**Figure 6:**
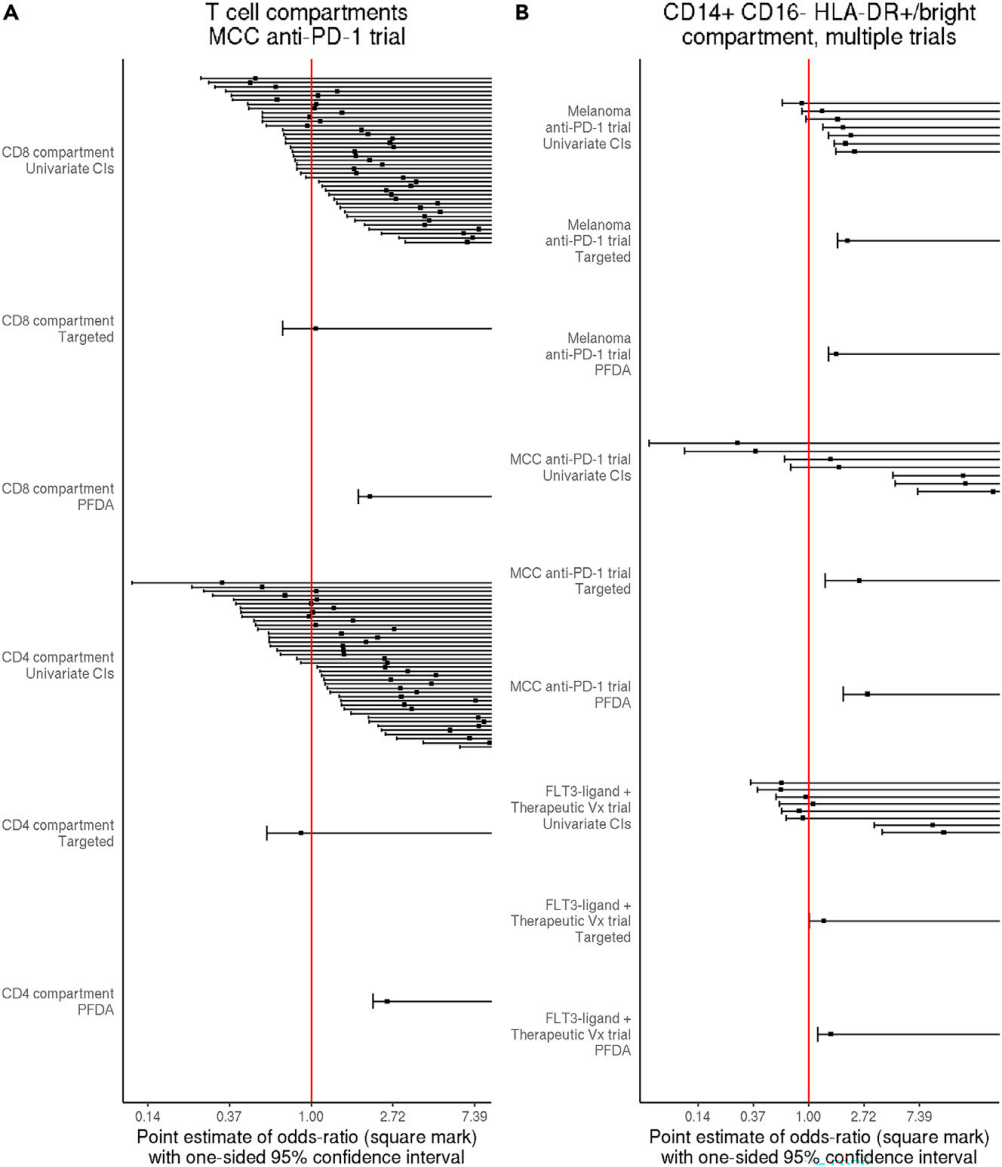
FAUST phenotypes enable cross-study meta-analysis of datasets stained with disparate marker panels (A) Forest plots displaying one-sided 95% confidence intervals (CIs) for increased abundance of CD3^+^ CD4^−^ CD8^+^ PD-1^dim/bright^ phenotypes (CD8 compartment) and CD3^+^ CD4^bright^ CD8^−^ PD-1^dim/bright^ phenotypes (CD4 compartment) in the MCC trial T cell panel. (B) Forest plots displaying one-sided 95% CIs for increased abundance of CD14^+^ CD16^−^ HLA-DR^+/bright^ CD3^−^ CD56^−^ CD19^−^ phenotypes in responders versus non-responders for three trials. Each panel shows CIs derived from fitting the univariate model to each FAUST phenotype consistent with the target, the 95% CI arising from the targeted approach, and the 95% CI derived by fitting a PFDA model jointly to the FAUST phenotypes and then testing for increased abundance using model coefficients.

To demonstrate how these approaches are used to test a specific hypothesis within a single study, we applied both of them to the T cell panel data from the MCC study (see “CD8+ T cells from virus-positive subjects correlate with in-tumor measurements; their association with outcome validates on independent data”). We hypothesized that total CD4 and total CD8 T cells expressing PD-1 were elevated in responders at baseline. To test this hypothesis with the targeted approach, we applied FAUST and extracted counts for all cells either with CD4^bright^ CD3^+^ CD8^−^ PD-1^bright^ or CD8^+^ CD3^+^ CD4^−^ PD-1^bright^ in their FAUST phenotypes. We then tested counts of these targeted phenotypes for increased abundance in responders using binomial GLMMs. To test the hypothesis with PFDA, we fitted two multivariate models to all FAUST phenotypes consistent with each of the targeted phenotypes, then used the model coefficients from each model to test for increased abundance in responding subjects. Using the targeted approach, we did not detect significantly increased abundance among responders in the targeted T cell phenotypes; using PFDA we did, since PFDA accounted for the heterogeneity of abundance patterns observed in the underlying FAUST phenotypes (Figure 6A).

These approaches can also be used to harmonize findings across different studies. To demonstrate this, we tested a second hypothesis that was generated from the unbiased FAUST analysis of several myeloid datasets. Both the MCC anti-PD-1 and FLT3-ligand + therapeutic Vx trials had cytometry data stained with a myeloid phenotyping panel. We also selected a myeloid phenotyping dataset from a previously published anti-PD-1 trial in metastatic melanoma.^9^ We used FAUST to conduct unbiased discovery on these three datasets and identified significant baseline phenotypes associated with clinical outcome at the Bonferroni-adjusted 10% level in all examined datasets.

In all considered studies, FAUST discovered significant phenotypes that were consistent with a principal finding of the previously published analysis:^9^ the frequency of CD14^+^ CD16^−^ HLA-DR^hi^ cells is associated with positive response to therapy (supplemental experimental procedures A2, A3, A4, and A.9). While the FAUST phenotypes were consistent with this finding, they were not directly comparable across trials due to differences in the staining panels. However, the panels shared a common group of markers: CD14, CD16, HLA-DR, CD3, CD56, and CD19. Based on this commonality, we hypothesized that CD14^+^ CD16^−^ HLA-DR^+/bright^ CD3^−^ CD56^−^ CD19^−^ cells exhibit increased abundance in responding subjects at baseline across the three examined trials.

To test this second hypothesis with the targeted approach, we applied FAUST and extracted counts of CD14^+^ CD16^−^ HLA-DR^+/bright^ CD3^−^ CD56^−^ CD19^−^ cells in each study and then tested for differential abundance using binomial GLMMs. To test this hypothesis with PFDA, we fit a multivariate model to the FAUST phenotypes containing the annotation CD14^+^ CD16^−^ HLA-DR^+/bright^ CD3^−^ CD56^−^ CD19^−^ in each trial and then tested for increased abundance using the estimated model coefficients. Using both the targeted approach and PFDA, we detected significantly increased abundance among responders in the CD14^+^ CD16^−^ HLA-DR^+/bright^ CD3^−^ CD56^−^ CD19^−^ phenotype (Figure 6B), consistent with the original publication.

The total CD4 and total CD8 T cell results demonstrate that the targeted approach does not always detect differential abundance when used to test phenotypes that contain numerous subpopulations with weak effects. On the other hand, PFDA can detect differential abundance in these cases by fitting a joint model to all FAUST phenotypes consistent with the target (so accounting for their correlation structure) and then using the model coefficients to test for an overall increase in abundance (Figure 6A). In the case of the CD14^+^ CD16^−^ HLA-DR^+/bright^ CD3^−^ CD56^−^ CD19^−^ results, there is less heterogeneity detected within the targeted phenotype across the studies, and both the top-down approach and PFDA detect increased abundance in responding subjects (Figure 6B). Together, these results demonstrate two ways that FAUST enables targeted hypothesis testing of pre-specified phenotypes, which in turn makes it possible to use FAUST to carry out validation analyses as well as to integrate findings across studies.

## Discussion

We applied FAUST to seven cytometry datasets (CyTOF and flow) generated from five independent studies described in the results section. We performed simulation studies which demonstrated that FAUST consistently recovers high-dimensional structures. We also performed a benchmark study on the FlowCAP-IV dataset which showed that FAUST discovered biologically predictive phenotypes. We used FAUST to conduct unbiased analysis of data from an MCC anti-PD-1 trial, discovered baseline correlates of response to therapy, demonstrated a novel way to visualize FAUST results, and showed how FAUST could be used to perform targeted validation of these correlates in independent data. We provided results showing how FAUST's phenotypes can be used to interrogate expected biological and technical signals in longitudinal studies. Finally, we illustrated how FAUST could be used to test pre-specified hypotheses using a targeted approach and PFDA.

Our simulation results show that FAUST's approach to discovery and down-selection captures genuine structure in data: FAUST did not severely over-partition the simulated datasets when the true number of clusters was small, nor did it under-partition as the true number of clusters grew (Figures 2A and 2C). In our experience, when we have applied FAUST to experimental cytometry datasets it is not uncommon for the number of down-selected phenotypes to be in the hundreds. These simulation results support the hypothesis that FAUST is identifying actual variation in such biological datasets, in favor of the competing hypothesis that FAUST is over-partitioning them as a methodological artifact. Moreover, the discovery results demonstrate that many methods are able to identify a predictive subset with good performance when we simulate a single perfect predictor of response (Figures 2B and 2D). However, they also demonstrate that when a method under-resolves the dataset it can cause the predictive subset to include many observations unrelated to the simulated response status, as measured by the adjusted Rand index (ARI) of the method's top cluster (Figure 2D). This raises the possibility that in the course of analyzing a real biological dataset, a method could identify a predictive subset of cells whose heterogeneity (due to under-resolution) masks the biological phenotype important for designing follow-on studies.

In our analysis of the MCC anti-PD-1 T cell dataset, we found that FAUST discovered hundreds of phenotypes and could be used to visualize them (see “FAUST identifies and visualizes baseline T cells in blood”). We also found that four of these phenotypes were significantly associated with clinical outcome in pre-treatment samples (see “CD8+ T cells from virus-positive subjects correlate with in-tumor measurements”). Notably, manual gating did not identify statistically significant T cell correlates of outcome in this study. We observed that the significant FAUST phenotypes involve combinations of PD-1, HLA-DR, and CD28 that were not investigated by the manual gating. We also applied FlowSOM, *k*-means, PARC, and PhenoGraph to this dataset. When we tested each method's clusters for association with outcome in baseline samples, we did not observe statistically significant correlates at either the Bonferroni or false-discovery-rate-adjusted 0.20 level (supplemental experimental procedures A.12). We were able to use FAUST to validate that the effector memory phenotypes discovered by FAUST in the MCC anti-PD-1 T cell dataset were also significantly increased in responding subjects in cryopreserved PBMCs from subjects in the same study, as well as the CyTOF dataset of Subrahmanyam et al.^49^ (see “CD8+ T cells from virus-positive subjects correlate with in-tumor measurements”).

Validation followed from the specificity of our hypotheses: we only tested three phenotypes of interest in the CyTOF dataset, while the authors reported examining 210 cell population fractions using FlowJo in the original analysis.^49^ Similar to the manual MCC analysis, none of the 210 cell populations manually examined in the original CyTOF analysis involved combinations of PD-1, HLA-DR, and CD28 (see Figures S2 and S4 in their publication).^49^ We believe that this is an important scientific feature of our method: when FAUST is used to perform unbiased discovery on datasets (such as in the MCC study), phenotypes that are found significant in subsequent modeling constitute testable hypotheses about specific cellular phenotypes. Furthermore, when independent datasets are publicly available (such as the CyTOF dataset of Subrahmanyam et al.^49^), we demonstrated that FAUST can be used to test and validate those hypotheses (see “CD8+ T cells from virus-positive subjects correlate with in-tumor measurements”).

The data reported here indicate that a population of effector memory CD4^+^ and CD8^+^ T cells co-expressing CD28, HLA-DR, and PD-1 are candidate biomarkers for response to pembrolizumab therapy in humans. These results are consistent with the findings of Kamphorst et al., who reported that HLA-DR^+^ CD28^+^ PD-1^+^ CD8 T cells (that also express CD38 and Ki-67) proliferate in the peripheral blood of non-small cell lung cancer (NSCLC) patients following PD-1 therapy (see Figure 4 of that publication).^42^ On the other hand, Ottonello et al. reported that the baseline frequency of PD-1^+^ EOMES^+^ CD8 T cells was significantly higher in NSCLC patients with progressive disease than in NSCLC patients with controlled disease prior to receiving nivolumab therapy.^52^ Contrasting this finding with our FAUST analysis suggests that the co-expression of CD28 and HLA-DR with PD-1 detected in the MCC study may be of particular importance in predicting positive response to therapy. Our findings also accord with literature reporting that CD28 signaling, when disrupted by PD-1, impairs T cell function.^41^ Brummelman et al. report an activated CD8^+^ T cell cluster enriched in lung tumor (see Figure 1D of that publication,^53^ cluster 7), whose phenotype (comparing markers that overlap between the panels) is consistent with the effector memory phenotypes described above in the MCC analysis (CCR7^−^, CD45RA^−^, CD25^−^, PD-1^dim/bright^, HLA-DR^+^). Whether the T cell populations discovered by FAUST are related to the reported T stem cell-like progenitor cells^54^ remains to be determined. Our data suggest that we may be capturing T cells in a state of dynamic transition.

Other blood-based candidate biomarkers for response to pembrolizumab therapy include: relative eosinophil count and relative lymphocyte count (both favorably associated with overall survival);^55^ CD69^+^ and MIP-1β^+^ natural killer cell subsets (increased in responding subjects);^49^ elevated levels of soluble PD-L1 (high pre-treatment levels were associated with increased likelihood of progressive disease);^56^ the observed ratio of Ki67^+^ CD8^+^ T cells to tumor burden at the post-treatment time point with peak T cell response (a higher ratio was associated with a better clinical outcome);^57^ and frequency of CD14^+^ CD16^−^ HLA-DR^hi^ monocytes (which predict progression-free and overall survival in response to anti-PD-1 immunotherapy).^9^ FAUST's detection of myeloid phenotypes as outcome correlates across three different datasets from three independent trials spanning different cancer types and therapies is also consistent with this last finding of Krieg et al.^9^

Overall, we demonstrated that FAUST can discover correlates of outcome in complex cytometry datasets that validate across independent trials and technologies. The reported correlates survived strong multiple testing adjustment and are biologically plausible (in that the FAUST phenotypes are consistent with related literature), which suggests that they represent real biological signals. We emphasize that additional prospective studies based on independent cohorts of patients with cancers (including MCC and melanoma) that have been approved for pembrolizumab therapy are needed to confirm both the T cell and myeloid signatures reported here.

There are several known limitations of the FAUST method. First, FAUST relies on one-dimensional statistics to detect multimodality and grow the forests used for annotation and discovery. Because of this, FAUST will not identify phenotypes if it does not detect marginal separation in any markers in a dataset. In our experience, this has not occurred when we have applied FAUST to heterogeneous populations of cells (e.g., lymphocytes) that have been profiled by a diverse marker panel. However, this condition could occur if FAUST is applied to a relatively homogeneous population of pre-gated cells (such as regulatory T cells) and the remaining markers in the panel do not exhibit multimodality. This situation is related to a second limitation of FAUST: if the method is applied to a large heterogeneous population of cells, markers in the panel which are known to be expressed solely on relatively rare subsets of cells will potentially be assigned a low depth score and dropped from discovery and annotation. If fluorescence-minus-one (FMO) controls exists, FAUST can be tuned to include the difficult-to-resolve markers by incorporating the location of each expression threshold informed by the controls into the analysis (see the marker boundary matrix in “FAUST method: tuning parameters” for more information).

Our results demonstrate that FAUST can consistently detect immunologically plausible candidate biomarkers from measurements made in blood using a simple, well-understood assay. They also demonstrate that FAUST may be of particular use in re-analyzing published datasets that have been distributed in public repositories since we applied FAUST to high-dimensional public CyTOF data as one way to validate the T cell correlates detected in the MCC study. Many large experimental cytometry datasets have already been published, and FAUST could be used to systematically analyze these datasets to produce standardized phenotypes. In turn, this could open the door to the re-analysis and meta-analysis of public datasets using approaches such as PFDA.

## Experimental procedures

### Resource availability

#### Lead contact

Requests for data and requests for additional information should be directed to the lead contact, Raphael Gottardo (raphael.gottardo@chuv.ch).

#### Materials availability

This study did not generate physical materials.

#### Data and code availability

Cytometry data from CITN-07 and CITN-09 analyzed in this study are available for download via DOI from figshare: 10.6084/m9.figshare.14791464. Cytometry data from the remaining studies were downloaded from FlowRepository:^48^

• FlowCAP-IV:^36^ https://flowrepository.org/id/FR-FCM-ZZ99
• Subrahmanyam et al.:^49^ https://flowrepository.org/id/FR-FCM-ZYKP
• Krieg et al.:^9^ https://flowrepository.org/id/FR-FCM-ZY34

FAUST is available as an R package at https://github.com/RGLab/FAUST. This implementation supports multithreading. When used, we have observed run times on large datasets comparable with phenograph^31^^,^^32^ (supplemental experimental procedures A.11). Scripts to reproduce the FlowCAP-IV analysis and the simulation study are available at

• https://github.com/RGLab/faust_flowcap4_analysis
• https://github.com/RGLab/faust_simulation_study

### FAUST method: Growing the annotation forest

To grow the annotation forest (Figure 1B), FAUST first tests each marker in each experimental unit for unimodality using the dip test.^23^ The hypothesis of unimodality is rejected for any marker that has dip test p values below 0.25. All markers which are deemed multimodal according to this dip criterion are then used to initialize gating strategies. Gate locations for each strategy are determined using the taut string density estimator.^58^ The location of each gate is the mid-point of any anti-modal component of the taut string. Since the taut string makes no assumptions about the number of modes present in a density, in principle this approach can lead to estimating an arbitrary number of gates in a given strategy. In practice, we only pursue strategies containing 4 or fewer gates under the assumption that marker expression of 5 or more expression categories does not reflect biological signal.

Once the initial set of gates are computed for a given marker, events are divided into subcollections relative to the gates for that marker, and the procedure recurses and repeats along each subcollection. Algorithm 1 describes the procedure. A gating strategy terminates when it meets any of the following stopping conditions. First, once a strategy involves any three combinations of markers, it terminates. This is because the space of gating strategies grows factorially with the number of markers. Due to this growth rate, nodes in the forest are penalized factorially relative to their depth in the gating strategy when we subsequently compute the depth score. Second, if at any point in a strategy FAUST fails to reject the null hypothesis of unimodality for all tested markers, the strategy terminates regardless of depth. Finally, a gating strategy terminates along a branch if all nodes along the branch contain too few cells. The algorithm displayed here assumes event measurements are distinct in the cytometry dataset, and all nodes in the forest contain in excess of 500 events. For details of how FAUST breaks ties and deals with nodes containing between 25 and 500 events, we refer the reader to Greene et al.^59^

Algorithm 1 **Grow annotation forest**

1: **function growAnnotationForest**(cells, depth, activeMarkers)
2: **if** (length(cells) < 500) or (depth >3) **then**
3: return strategy ⊳ Gating strategy stops due to depth, event
constraints.
4: **else**
5: newDepth←depth+1
6: multimodalList←empty list
7: **for** mIdx∈(columns(exprsMatrix)∩activeMarkers) **do**
8: **if** pValue(dipTest(exprsMatrix[currentCells,mIdx]))<0.25 **then**
9: append(multimodalList,mIdx)
10: **if** length(multimodalList)==0 **then**
11: return strategy⊳Gating strategy stops due to shape constraint.
12: **else**
13: **for** mIdx in multimodalList **do**
14: boundaryList←empty list
15: ComputetautstringdensityestimateofexprsMatrix[cells,mIdx]
16: boundaryList←mid-points of antimodal components of taut string
17: remainingMarkers←activeMarkers∖mIdx
18: **for** iin[1,length(boundaryList)] **do**
19: lg←boundaryList[(i-1)]
20: ug←boundaryList[i]
21: newCells←rowsofexprsMatrix[cells,mIdx]betweenlgandug
22: growAnnotationForest(newCells, newDepth,remainingMarkers)

### FAUST method: Depth-score computation

While we were aware of tree-based learning algorithms when developing this score (in particular, random forests,^60^ a related unsupervised approach using well-chosen synthetic data,^61^ and related measures of variable importance),^62^^,^^63^ here we chose to develop a score that attempts to incorporate characteristics of the non-parametric tools used to grow the annotation forest (in particular the dip test^23^ and taut string).^58^ Suppose there are p>1 active markers in a sample. To compute the depth score for any of the *p* markers, the annotation forest is first examined to determine the following quantities: $d_{1}$, the number of times different markers are gated in the root population; $d_{2}$, the number of times children of the root are gated; and $d_{3}$, the number of times grandchildren of the root are gated. For i∈{1,2,3}, define

$$\delta_{i}\equiv \frac{1}{d_{i}}\text{.}$$

For 1≤m≤p, let

$$N_{m}\equiv \left\{N_{m,1},N_{m,2},\ldots ,N_{m,n}\right\}\;\;$$

be the set of all *n* parent nodes in the annotation forest for which the null hypothesis of unimodality is rejected for marker *m*. For a parent node 1≤j≤n, let $1_{R}$ denote the indicator function that is 1 when $N_{m,\;j}$ is the root population. Similarly, let $1_{C}$ denote an indicator of a child of the root, and $1_{G}$ a grandchild of the root. Define the scoring function

$$Q\left(N_{m,\;j}\right)\equiv \left(1-\alpha_{R}\right)1_{R}\left(N_{m,\;j}\right)+\left(1-\alpha_{R}\right)\left(1-\alpha_{C}\right)1_{C}\left(N_{m,\;j}\right)+\left(1-\alpha_{R}\right)\left(1-\alpha_{C}\right)\left(1-\alpha_{G}\right)1_{G}\left(N_{m,\;j}\right)\text{, }$$

where, abusing notation, we let

$$\alpha_{R}\equiv \alpha_{R}\left(N_{m,\;j}\right)$$

$$\equiv \text{the dip test p}-\text{value in the root population of the gating strategy that led to }N_{m,\;j}\text{.}$$

We allow $\alpha_{C}$ and $\alpha_{G}$ to be defined similarly. The function *Q* can be interpreted as a measure of the quality of the gating strategy that led to node $N_{m,\;j}$. In the case of a grandchild node that had clear modal separation along all markers in the strategy, $Q\left(N_{m,\;j}\right)\approx 1$, while a grandchild node that had p values of 0.25 at each ancestral node, $Q\left(N_{m,\;j}\right)\approx 27/64=0.75^{3}$.

Let $P_{m}$ be the population size for marker *m* in the root population. Next, define

$$P\left(N_{m,\;j}\right)\equiv \frac{\#\text{ of cells in node }N_{m,\;j}}{P_{m}}\text{.}$$

Finally, define

$$D\left(N_{m,\;j}\right)\equiv \delta_{1}\cdot 1_{R}\left(N_{m,\;j}\right)+\delta_{2}\cdot 1_{C}\left(N_{m,\;j}\right)+\delta_{3}\cdot 1_{G}\left(N_{m,\;j}\right)\text{.}$$

The depth score is defined to be the normalized sum

(Equation 4.1) $$DS\left(N_{m}\right)\equiv \frac{\sum_{i=1}^{n}Q\left(N_{m,\;i}\right)\cdot P\left(N_{m,\;i}\right)\cdot D\left(N_{m,\;i}\right)}{{\text{max}}_{1\leq q\leq p}DS\left(N_{q}\right)}\equiv \frac{\sum_{i=1}^{n}\omega \left(N_{m,\;i}\right)}{{\text{max}}_{1\leq q\leq p}DS\left(N_{q}\right)}\text{.}$$

The depth score maps $N_{m}$ into [0,1], with at least one marker in a gated sample achieving the maximal score of 1. This is taken as a measure of separation quality: the best scoring marker according to the depth score is taken to be the best separated marker in that sample at the root population, and conditionally along all other gating strategies. Normalizing to the unit interval allows depth scores to be compared across experimental units for given markers. By using the factorial weights $\delta_{i}$, the depth score also explains why FAUST only explores gating strategies involving, at most, combinations of three markers in its scoring and marker selection phase. Adding more combinations of markers induces a factorial increase in computational cost. But any marker that enters a gating strategy at depth 4 (or beyond) will be dominated in depth score by those markers initially gated in the annotation forest at or near the root population. Consequently, after normalization in experiments with a large number of markers, such markers have a depth score an *ε* above zero, and are effectively never selected by FAUST for discovery and annotation—hence the restriction to 3-marker gating strategies.

### FAUST method: Annotation boundary estimation

The depth score is also used to estimate annotation boundaries. Recalling FAUST only explores gating strategies with 4 or fewer annotation boundaries, FAUST partitions the set

$$N_{m}=G_{1}\cup G_{2}\cup G_{3}\cup G_{4}\text{.}$$

Define

$$G_{1}\equiv \left\{N_{m,\;i}\in N_{m}\;|N_{m,\;i}\text{ has a single gate determined by the taut string}\right\}\text{.}$$

$G_{2},G_{3},$ and $G_{4}$ are defined similarly. In other words, each $G_{i}$ is the subset of nodes in the annotation forest for marker *m* with *i* gates. Recalling Equation 4.1 (which defines ω), we can partition the score sum

$$\sum_{i=1}^{n}\omega \left(N_{m,\;i}\right)=\sum_{j=1}^{4}\sum_{N\in G_{j}}\omega \left(N\right)\text{.}$$

FAUST selects the number of annotation boundaries for the marker *m* by choosing the set $G_{j}$ with the maximal sum $\underset{N\in G_{j}}{\sum }\omega \left(N\right)$. Letting $g_{1}\left(N_{m,\;j}\right)$ denote the smallest gate location estimated by the taut string in node $N_{m,\;j}$ (which is the only gate location if FAUST selects $G_{1}$), FAUST estimates the phenotypic boundary locations for the marker by taking the weighted average

$$\frac{\sum_{N\in G_{j}}\omega \left(N\right)g_{1}\left(N\right)}{\sum_{N\in G_{j}}\omega \left(N\right)}\text{.}$$

In the event FAUST selects $G_{j},\;j>1$ (i.e., multiple annotation boundaries), similar weighted averages are taken for $g_{2}\left(N_{m,j}\right)$, and so forth.

### FAUST method: Marker selection

Markers are selected by comparing the empirical depth-score selection quantile across experimental units to a depth-score selection threshold value. All markers whose empirical quantile exceeds the threshold are used for discovery and annotation. Both values are tunable.

### FAUST method: Boundary standardization

FAUST standardizes the number of annotation boundaries for each marker by majority vote. The most frequently occurring number of annotation boundaries across experimental units is chosen as the standard number. This behavior can be modified via the preference list tuning parameter (see supervised boundary estimation list) in order to incorporate prior biological information into FAUST.

Next, for a given marker, FAUST selects the set of experimental units where the number of annotation boundaries for that marker matches the standard. Then, by rank, FAUST computes the median location of each phenotypic boundary across experimental units. We refer to these median boundary locations as the standard boundaries.

FAUST enforces standardization of annotation boundaries for non-conforming experimental units by imputation or deletion. Imputation in an experimental unit occurs when FAUST estimates fewer boundaries than the standard. In this case, each boundary in the non-conforming unit is matched to one of the standards by distance. Unmatched standards are used to impute the missing boundaries. Similar distance computations are done in the case of deletion, but FAUST deletes boundaries that are farthest from the standards. For both imputation and deletion, if multiple boundaries match the same standard, then the boundary minimizing the distance is kept and the other boundaries are deleted. Should this result in standards that do not map to any boundaries, those unmatched standards are used to impute the missing boundaries.

If the user modifies the imputation hierarchy parameter (see tuning parameters), the previously described imputation process is modified in the following way. First, FAUST iterates over distinct values of the imputation hierarchy setting, and will attempt to impute missing boundaries for an experimental unit by only using data from experimental units with the same setting. Similarly, deletions occur only using data from experimental units with the same imputation hierarchy setting. Once this is complete, FAUST will then impute missing boundaries and delete excess boundaries using data across all experimental units.

### FAUST method: Phenotype discovery and cluster annotation

For each experimental unit, FAUST constructs a forest of partition trees (randomly sampled) and annotates selected leaves from this forest relative to the standardized annotation boundaries. Partition tree construction is similar to tree construction for the annotation forest (see growing the annotation forest), but they are not depth constrained: a tree continues to grow following the previously described strategy until each leaf is unimodal according to the dip test^23^ or contains fewer than 25 cells. Consequently, a single partition tree defines a clustering of an experimental unit. Clusterings from the forest of partition trees are combined into a single clustering in the following manner. To ensure cells are not assigned to multiple clusters, a subset of leaves of the partition forest are selected by scoring leaves according to shape criteria and then selecting a subset of leaves across partition trees that share no cells to maximize their total shape score. The shape score is defined in terms of trimmed sample L moments^64^^,^^65^ as well dip test p values^23^ of leaf margins. Only the selected leaves are given phenotypic annotations. Phenotypes are determined for a selected leaf by comparing the median of each margin with the standardized annotation boundary for the sample, then assigning the leaf a label that locates the leaf relative to the sample boundaries. In the case of a single boundary for a phenotype, the location then maps to “−” or “+;” for two boundaries, “−,” “dim,” and “bright.” FAUST keeps a list of discovered phenotypes for each experimental unit and concludes by returning exact counts of cells in each sample whose phenotypes exceed a user-specified occurrence frequency threshold. For more details of the scoring and selection procedure, we refer the reader to Greene et al.^59^

### FAUST method: Tuning parameters

We describe here the key tuning parameters of FAUST.

#### Experimental unit

This parameter is used to link individual samples together into a single experimental unit. All samples with the same “experimental unit” value are concatenated prior to FAUST conducting discovery and annotation.

#### Imputation hierarchy

This parameter is used to link experimental units together during the standardization of annotation thresholds. All experimental units with the same “imputation hierarchy” value will define a class of units across which the boundary standardization process first occurs. For example, suppose a study contains tissue samples and blood samples stained by the same marker panel. The imputation hierarchy can be used so that if a boundary is missing for a marker from a blood sample, FAUST will impute the missing boundary using boundaries from other blood samples if possible. Similarly, if a boundary is missing for a marker from a tissue sample, this parameter will have FAUST estimate the missing boundary location using other tissue samples alone if possible. If it is not possible to impute a missing boundary within a class (since the boundary is never estimated with the class), imputation then occurs across all experimental samples. By default, this parameter is flat: standardization occurs across all experimental samples unless otherwise specified by the user.

#### Starting cell population

The name of the population in the manual gating strategy where FAUST conducts discovery and annotation.

#### Active markers

A list of all markers in the experiment that can possibly be used for discovery and annotation in the starting cell population. FAUST will only compute the depth score for markers in this initial set.

#### Marker boundary matrix

A 2×n matrix of lower and upper protein expression bounds. When the manual gating strategy does not remove all debris or doublets from the starting cell population, samples can appear to have clusters of events along at very low or very high expression values for some markers. By setting boundaries for those markers to exclude these doublet or debris clusters, FAUST treats all events below the lower and above the upper bounds as default low or high, respectively. These events are not dropped from the experiment. However, they are ignored when testing for multimodality and subsequent density estimation. The number of events in a marker that falls between the lower and upper marker boundaries in the starting cell population defines the effective sample size for that marker. By default, values in the marker boundary matrix are determined as follows: the 1st and 99th percentiles of expression by marker are computed per sample, and the fifth percentile of first percentiles and the 95th percentile of 99th percentiles are then used as the bounds for a marker. If FMO controls exist for a flow cytometry experiment, the lower bound for a given marker can be modified so that the lower boundary is near the expression threshold contained in the corresponding control: this can help FAUST include hard-to-resolve markers of interest. An example of how tuning this parameter affects the distribution of annotation thresholds is given in supplemental experimental procedures A.8.

#### Depth-score selection quantile

The empirical quantile of a marker's depth score across all experimental units that is used to compare against a user-selected depth-score threshold. By default, this parameter is set to the median.

#### Depth-score selection threshold

A value in [0,1] used to select a subset of markers to be used in discovery and annotation based on their empirical depth-score selection quantile. By default, this parameter is set to 0.01.

#### Supervised boundary estimation list

This allows the user to modify FAUST's default gate standardization methodology for each marker. This parameter is one way to incorporate prior (biological) knowledge in the FAUST procedure: if a marker is known to have a certain range of expression, such as low-dim-bright, this can be used to encourage or force FAUST to estimate the corresponding number of annotation boundaries from the data. Similarly, if FMO controls have been collected for a marker, this parameter can be used to set the phenotypic boundary according to the controls.

#### Phenotype occurrence threshold

An integer value (set to 1 by default) used to include or exclude discovered phenotypes in the final count matrix returned by FAUST. If a phenotype appears at least phenotype occurrence threshold times across experimental units, it is included in the final counts matrix. If an experiment contains 4 or fewer samples, FAUST includes all discovered phenotypes by default. If an experiment contains more than 4 samples, FAUST sets this parameter by computing the greatest convex minorant (GCM) of the distribution of occurrences over all phenotypes, for phenotypes observed between phenoMin≡max(2,0.05⋅number of samples) and phenoMax≡min(number of samples−1,0.95⋅number of samples) times. FAUST then finds the knot on the GCM that maximizes the distance between the GCM and the line connecting phenoMin and phenoMax, taking this as an estimate of the elbow of the GCM. Phenotypes exceeding the threshold are the consistently detected phenotypes and are included in the annotated count matrix produced by FAUST.

### FAUST algorithm: Annotation embedding

Given a completed FAUST run, an annotation embedding is generated for a sample by first winsorizing the observed expression data for each selected marker to the 1st and 99th percentile. Samples from N(0,0.01) are taken to perturb the winsorized data at the 1st and 99th percentile in order to break ties in rare annotation groups in the sequel. The winsorized data is then transformed as follows. For each FAUST annotation observed in the dataset (which includes phenotypic annotations removed via down-selection), all rows in the winsorized dataset corresponding to the specific FAUST annotation are selected. The observed expression of each subset comprises an annotation grouping, and is scaled to have mean zero and unit standard deviation for each marker. The maximum and minimum values of each annotation grouping are both recorded.

For each marker, the maximum and minimum values within each distinct annotation label are used to determine a final scaling value. In addition, for each marker an equispaced grid is established as landmarks for the standardized data. For each annotation grouping, the standardized expression data are translated for each marker to the corresponding landmark and scaled to by the final scaling factors. This ensures the annotation groupings translated to distinct landmarks for a given marker do not overlap. Once all annotation groupings are translated and scaled, the dataset has been prepared for annotation embeddings. This can be performed using any dimensionality reduction algorithm; by default, we use the UMAP algorithm with “qualitative” parameter settings.^44^

### Simulation study: Estimating the number of clusters and partition scoring

The number of clusters simulation is designed as follows. A reference probability vector with 1 component set to 0.1425, 2 components set to 0.07125, 4 to 0.035625, 8 to 0.0178125, 16 to 0.00890625, 32 to 0.004453125, and 64 to 0.002226562 is initialized in decreasing sort order. In the simulation, the number of clusters is varied from 5 to 115 components by step size of 10, and the simulation repeats 5 times for each setting.

For a given number of clusters, mixture weights for a multivariate Gaussian mixture model are determined by selecting the subset of the reference probability vector (starting from the first position) corresponding to simulated number of clusters. If the subset of weights does not sum to 1, residual mass is uniformly added to all elements of the subset so that they sum to unity. The number of components in the mixture model is identified as the true number of clusters in the simulation. For each simulation iteration a dataset of 10 independent samples is generated, with each sample consisting of 25,000 observations drawn from a 10-dimensional multivariate Gaussian mixture with the specified number of components.

Before generating the samples, a fixed collection of mean vectors $\mu_{c}$, 1≤c≤10 is determined for the Gaussian mixture components. Each of the 10 entries of $\mu_{c}$ are randomly selected from the columns of Table 1 and represent whether or not the simulated variable is expressed. When an entry of $\mu_{c}$ is from the “Not expressed” row of Table 1, the corresponding variable is labeled “−.” Similarly, when an entry of $\mu_{c}$ is from the “Expressed” row of Table 1, the corresponding variable is labeled “+.” As an example, the annotation “V1 − V2 − V3 + V4 − V5 + V6 − V7 − V8 − V9 + V10 −” indicates that the mean vector $\mu_{c}$ of the mixture component contains 0 for V1, V2, V4, V6, V7, V8, and V10, while it is 8 for V3, V5, and V9. Each mean vector is associated with an element of the mixture weight vector.

**Table 1:** Possible mean vector entries for the 10 simulation variables

	V1	V2	V3	V4	V5	V6	V7	V8	V9	V10
Not expressed	0	0	0	0	0	0	0	0	0	0
Expressed	8	8	8	8	8	8	8	8	8	8

Covariance matrices ${\text{Σ}}_{c}$ are randomly sampled; they are always constrained to have variances between 1 and 2, but otherwise are randomly generated sample by sample and component by component. The number of observations sampled for each component is determined by taking a single sample from a multinomial distribution, with multinomial parameters set using the weight vector and to ensure 25,000 total observations per sample. Sample-to-sample variability is modeled during the simulation by modifying the covariance matrices as previously described, and perturbing each component's mean vector in the following way: 10 samples are taken from a mean 0 Gaussian with standard deviation set to $1/\sqrt{2}$, rounded to the nearest integer, and used to translate the mean vector.

Methods are then applied to the simulated dataset. Every method (except FAUST, see the following) is applied to the 10 samples concatenated together. Methods are applied with the following parameter settings.

• FAUST is run with default analysis parameter settings. This means that the number of clusters is estimated automatically, marker selection is done automatically, and the experimental unit is set to individual samples. The only change from default is that FAUST is set to use 10 threads in order to accelerate the computation.
• DEPECHE^27^ was run with *k* parameter (the number of initial cluster centers) set to 2⋅simulated true number of clusters, and was allowed 10 threads to accelerate the computation.
• flowMeans^10^^,^^28^ is run with default parameter settings, except that the MaxN parameter is set to twice the simulated true number of clusters to accelerate the computation.
• FlowSOM^6^^,^^29^ (automated) is run with default parameter settings, except that the maxMeta parameter is set to 90. We did not scale with the number of clusters due to FlowSOM's dependency on ConcensusClusterPlus: in our simulation runs where the number of clusters exceeded 100, this led to an error in an internal call of stats:cutree with the *k* > 9 parameter causing an error.
• FlowSOM^6^^,^^29^ (oracle) is run using the default parameters of the FlowSOM:BuildSOM function with the grid parameters set to xdim = 1 and ydim = simulated true number of clusters. This grid topology is selected as per the self-organizing map discussion in pages 421–423 of Hennig et al.^66^
• *k*-means was run using the simulated true number of clusters for *k*, for 10,000 iteration maximum, using the Lloyd algorithm.
• PARC^30^ was run with default parameters via the reticulate R package.^67^
• PhenoGraph^31^^,^^32^ was run with default parameter settings.
• Rclusterpp^33^ was run with default parameter settings. The k parameter in cutree was set to min(true number of clusters,nrow(rcppResult$merge)+1) to guarantee a successful run for each simulation iteration.

For each method, the number of clusters estimated is recorded (if the method did not require the simulated truth as a tuning parameter). For FAUST, the number of clusters was identified as the number of down-selected phenotypes in the final count matrix. Additionally, the adjusted Rand index (ARI) was computed for each clustering relative to the simulated truth, both on the entire dataset as well as the subset of observations annotated by FAUST (the down-selected phenotypes). For FAUST, all events that were not annotated as one of the down-selected phenotypes were treated as a single class: such events were given the annotation “0_0_0_0_0” to indicate that no down-selected phenotype was applied.

### Simulation study: Predicting simulated responder status

This simulation begins by fixing a weight vector with 125 components, listed in Table 2. By design, the weight of component 120 is set to 0.0011, and this component is designated as the perfect predictor of response status. For responding samples, the weight vector is modified to the values listed in Table 3. Comparing the responding weight vector in Table 3 with the non-responding weight vector in Table 2, we see that the predictive component is exactly doubled. We also see that the other 124 weights are proportionally modified to achieve that doubling. This proportional modification was done to break any correlation between the other 124 components and responder status: in expectation, there will be differential abundance in the predictive component alone.

**Table 2:** Fixed weight vector for non-responding samples

0.16155	0.0712	0.0712	0.0356	0.0356	0.0356	0.0356	0.0178	0.0178	0.0178	0.0178	0.0178	0.0178
0.0178	0.0178	0.0089	0.0089	0.0089	0.0089	0.0089	0.0089	0.0089	0.0089	0.0089	0.0089	
0.0089	0.0089	0.0089	0.0089	0.0089	0.0089	0.0045	0.0045	0.0045	0.0045	0.0045	0.0045	0.0045
0.0045	0.0045	0.0045	0.0045	0.0045	0.0045	0.0045	0.0045	0.0045	0.0045	0.0045	0.0045	
0.0045	0.0045	0.0045	0.0045	0.0045	0.0045	0.0045	0.0045	0.0045	0.0045	0.0045	0.0045	0.0045
0.0022	0.0022	0.0022	0.0022	0.0022	0.0022	0.0022	0.0022	0.0022	0.0022	0.0022	0.0022	
0.0022	0.0022	0.0022	0.0022	0.0022	0.0022	0.0022	0.0022	0.0022	0.0022	0.0022	0.0022	0.0022
0.0022	0.0022	0.0022	0.0022	0.0022	0.0022	0.0022	0.0022	0.0022	0.0022	0.0022	0.0022	
0.0022	0.0022	0.0022	0.0022	0.0022	0.0022	0.0022	0.0022	0.0022	0.0022	0.00209	0.00198	0.00187
0.00176	0.00165	0.00154	0.00143	0.00132	0.00121	0.0011	0.0011	0.0011	0.0011	0.0011	0.0011	

The predictive component's weight is 0.0011 (the first such entry in the table). The weight vector is specified left-to-right, top-to-bottom, with empty cells in the table skipped.

**Table 3:** Fixed weight vector for responding samples

0.16120344	0.07105221	0.07105221	0.03553053	0.03553053	0.03553053	0.03553053	0.01776969	0.01776969	0.01776969	0.01776969	0.01776969	0.01776969
0.01776969	0.01776969	0.00888927	0.00888927	0.00888927	0.00888927	0.00888927	0.00888927	0.00888927	0.00888927	0.00888927	0.00888927	
0.00888927	0.00888927	0.00888927	0.00888927	0.00888927	0.00888927	0.00449895	0.00449895	0.00449895	0.00449895	0.00449895	0.00449895	0.00449895
0.00449895	0.00449895	0.00449895	0.00449895	0.00449895	0.00449895	0.00449895	0.00449895	0.00449895	0.00449895	0.00449895	0.00449895	
0.00449895	0.00449895	0.00449895	0.00449895	0.00449895	0.00449895	0.00449895	0.00449895	0.00449895	0.00449895	0.00449895	0.00449895	0.00449895
0.00220401	0.00220401	0.00220401	0.00220401	0.00220401	0.00220401	0.00220401	0.00220401	0.00220401	0.00220401	0.00220401	0.00220401	
0.00220401	0.00220401	0.00220401	0.00220401	0.00220401	0.00220401	0.00220401	0.00220401	0.00220401	0.00220401	0.00220401	0.00220401	0.00220401
0.00220401	0.00220401	0.00220401	0.00220401	0.00220401	0.00220401	0.00220401	0.00220401	0.00220401	0.00220401	0.00220401	0.00220401	
0.00220401	0.00220401	0.00220401	0.00220401	0.00220401	0.00220401	0.00220401	0.00220401	0.00220401	0.00220401	0.00209425	0.0019845	0.00187474
0.00176498	0.00165522	0.00154546	0.00143571	0.00132595	0.00121619	0.0022	0.00110643	0.00110643	0.00110643	0.00110643	0.00110643	

The predictive component's weight is exactly 0.0022. The weight vector is specified left-to-right, top-to-bottom, with empty cells in the table skipped.

For each simulation iteration, 20 samples of 25,000 observations are generated according to the procedure described in “estimating the number of clusters and partition scoring.” Ten of the 20 samples are randomly assigned as responders and simulated using the responder weight vector in Table 3. The remaining 10 samples are designated non-responders and are simulated using the non-responder weight vector in Table 2.

For each simulation iteration, each method described in “estimating the number of clusters and partition scoring” (with the exception of flowMeans) is applied to the simulated dataset. For each method, a binary clustering identifying each discovered cluster is determined. The ARI of each binary clustering relative to a binary clustering identifying the simulated predictive component is then computed. Additionally, for each method, a per-sample count matrix of the resulting clustering is derived, with rows corresponding to simulated samples, columns to a cluster, and cells to the count of the cluster in a sample. Using this count matrix, each cluster is tested for association with responder status using a binomial GLMM with sample-level random effect (this is the model used in our analysis of real cytometry datasets, specified in Equation 4.2). The cluster with the smallest p value is designated the “top cluster” discussed in “FAUST resolves high-dimensional structure in simulation studies.” The binary ARI for this cluster is recorded. Then, using the per-sample frequency of this “top cluster,” the 5cvAUC using a logistic model predicting responder type given frequency is computed, and each simulation iteration is recorded.

In addition to the previously described methods, we also apply the following approaches with specified parameter settings and analysis modifications for each simulation iteration.

• Citrus^5^ analysis conducted using the citrus.full function with parameters set to default for modeling, and to ensure all data were used in the analysis. For our version, the defaults were the “family” parameter set to “classification,” the “modelTypes” parameter set to “glmnet,” the “featureType” set to “abundances,” the “nfold” parameter set to “1,” the “fileSampleSize” set to “25,000,” the “transformColumns” parameter set to “NULL,” the “transformCofactor” parameter set to “NULL,” and the “scaleColumns” parameter set to “NULL.” All differentially abundant clusters defined by citrus that minimize the cross-validation are taken together as the “top cluster.” This means that for citrus alone, on a simulation iteration we potentially use frequencies from multiple clusters to compute the 5cvAUC, and that the ARI relative to the simulated predictive element is computed using a single binary cluster that possibly consists of multiple clusters.
• diffCyt^19^^,^^34^ is applied using the diffcyt function in R, with the grid parameters set to xdim = 11 and ydim = 12 and analysis type set to “DA” for differential abundance. This produces a rectangular grid of 131 clusters, which slightly over-partitions the 125 simulated true number of clusters. We defined “top cluster” for diffCyt as the first element of the significance table generated by the function diffcyt:topTable. For this cluster only, an ARI relative to the simulated predictive element and a 5cvAUC is computed each simulation iteration.
• FlowSOM^6^^,^^29^ (over-partitioned) using the default parameters of the FlowSOM:BuildSOM function with the grid parameters set to xdim = 15 and ydim = 15. This produces an over-partitioned grid of 225 clusters (relative to the true 125). ARI and 5cvAUC values are computed in the same fashion as the other methods.

### CITN-09 T cell panel analysis

The CITN-09 T cell staining panel is described in supplemental experimental procedures A.25. FAUST tuning parameter settings for this dataset are described in supplemental experimental procedures A.18.

Between 1 and 4 samples were collected from 27 patients with stage IV and unresectable stage IIIB Merkel cell carcinoma (MCC)^38^^,^^68^ spanning the course of treatment. All 27 patients had samples collected at baseline (cycle C01, before initiation of anti-PD-1 therapy); 16 at cycle C02 (3 weeks post-treatment of the second cycle of therapy); 22 at cycle C05 (12 weeks post-treatment of the fifth cycle of therapy); and 13 at end of trial (EOT, patient specific). This produced a dataset consisting of 78 samples in total. Eighteen of 27 subjects responded to therapy (CR/PR) for an observed response rate of 67%. Each sample was pre-gated to remove debris and identify live lymphocytes.

FAUST was applied to the subset of live lymphocytes with the experimental unit set to individual patient samples. After tuning, FAUST selected the markers CCR7, CD127, CD25, CD28, CD3, CD45RA, CD4, CD8, HLA-DR, and PD-1 for the discovery and annotation of phenotypes. In the 27 baseline samples, FAUST phenotypes annotated CD3^+^ were tested for association with response to therapy (CR/PR) using a binomial GLMM with a subject-level random effect (the model is formally specified in Equation 4.2).

At Bonferroni-adjusted 0.10 level, four significant phenotypes (discussed in “FAUST identifies and visualizes baseline T cells in blood associated with outcome in CITN-09” and “CD8+ T cells from virus-positive subjects correlate with in-tumor measurements”) were detected. These phenotypes and their adjusted p values are listed in Table 4.

**Table 4:** The four significant phenotypes at the Bonferroni-adjusted 0.10 level discovered and annotated by FAUST in the analysis of the CITN-09 T cell panel

FAUST phenotype	Bonferroni p value
CD4^bright^ CD8^−^ CD3^+^ CD45RA^−^ HLA-DR^−^ PD-1^dim^ CD28^+^ CD127^−^ CD25^−^ CCR7^−^	0.0016
CD4^−^ CD8^+^ CD3^+^ CD45RA^−^ HLA-DR^+^ PD-1^dim^ CD28^+^ CD127^−^ CD25^−^ CCR7^−^	0.0072
CD4^−^ CD8^+^ CD3^+^ CD45RA^−^ HLA-DR^+^ PD-1^bright^ CD28^+^ CD127^−^ CD25^−^ CCR7^−^	0.0487
CD4^bright^ CD8^−^ CD3^+^ CD45RA^−^ HLA-DR^+^ PD-1^dim^ CD28^+^ CD127^−^ CD25^−^ CCR7^−^	0.0599

To specify the binomial model, let $c_{i,\;k}$ denote the number of events in FAUST cluster *k* for sample *i*. Let $n_{i}$ denote the number of events annotated as CD3^+^ by FAUST in the *i*^th^ subject's baseline sample. Similar to Nowicka et al.,^39^ we assume $c_{i,\;k}∼\text{Binomial}\left(n_{i},\;\mu_{i,\;k}\right)$. Our model is

(Equation 4.2) $${\text{logit}}^{-1}\left(\mu_{i,\;k}\right)=\beta_{0}+\beta_{1}\cdot \text{Responder}+\xi_{i,\;k}\text{,}$$

where Responder is an indicator variable equal to 1 when the subject exhibits CR or PR to therapy, and 0 otherwise, and each $\xi_{i,k}∼N\left(0,\sigma_{i,k}^{2}\right)$ is a subject-level random effect. The R package lme4 was used to fit all GLMMs.^69^

### CITN-09 cryopreserved PBMC analysis

The staining panel used for the CITN-09 cryopreserved PBMC analysis is the same as that used in the initial T cell analysis, and is described in supplemental experimental procedures A.25. FAUST tuning parameter settings for this dataset are described in supplemental experimental procedures A.19.

FAUST was used to generate annotation thresholds in 15 cryopreserved PBMC patient samples from the CITN-09 MCC study. As in the initial discovery analysis, FAUST was applied to the subset of live lymphocytes with the experimental unit set to individual patient samples. FAUST was tuned to generate thresholds for the markers CCR7, CD127, CD25, CD28, CD3, CD45RA, CD4, CD8, HLA-DR, and PD-1, those selected in the initial analysis. These thresholds were used to derive counts for CD4 and CD8 phenotypes comparable with those discovered in the initial analysis. The resulting counts were tested for differential abundance between responders (PR/CR) and non-responders (PD/SD) in virus-positive subjects for the CD8^+^ phenotype, and between all responders and non-responders in the CD4^+^ phenotypes.

### FlowCAP-IV analysis

The HIV progression dataset used in FlowCAP-IV was downloaded from FlowRepository, https://flowrepository.org/id/FR-FCM-ZZ99. FloReMi scripts submitted for the FlowCAP-IV analysis were downloaded from the github repository https://github.com/SofieVG/FloReMi. The first script 1_preprocessing.R was modified to respect the local file system (but otherwise unchanged) and then used to pre-gate live T cells from the FlowRepository data.

FAUST was first used to perform discovery and annotation of phenotypes on the pre-gated live T cells in the 382 samples constituting the training set. The tuning parameter setting used in the FAUST analysis of the training set is described in supplemental experimental procedures A.23. After tuning, FAUST selected the markers CCR7, CD154, CD27, CD45RO, CD4, CD57, and CD8 for phenotype discovery and annotation. FAUST was then used to generate annotation thresholds on the 384 samples comprising the test set for these 7 markers. The tuning parameter setting used in the FAUST analysis of the test set is described in supplemental experimental procedures A.24. Once thresholds were generated for the test set, counts for the phenotypes discovered on the training set were derived on the test set relative to the thresholds.

For predictive modeling, we only used the random survival forest,^70^ the best-performing model in the initial report.^37^ Two classes of features were used to fit the model on the training set. The FAUST phenotypes could be differentiated into those annotated CD154^−^ and those annotated CD154^+^. Since CD154 is an activation marker,^71^ the features admitted to the model were the frequencies of all phenotypes annotated CD154^−^ in unstimulated samples, and the difference in frequency between stimulated and unstimulated samples for all phenotypes annotated CD154^+^. These features were used to fit the model on the training set, with parameter settings for the random survival forest taken from the script 4b_randomSurvivalForest.R (again found in the repository https://github.com/SofieVG/FloReMi). The reported concordance and −log_10_(p value) were computed, as in the initial report, from fitting a Cox proportional hazard model to the fitted values on the test set using the survival R package.^72^^,^^73^

### CITN-09 myeloid panel

The CITN-09 myeloid staining panel is described in supplemental experimental procedures A.26. FAUST tuning parameter settings are described in supplemental experimental procedures A.20. This dataset consisted of 69 samples stained to investigate myeloid cells. An initial screen comparing the ratio of the number of events in the singlet gate with the number of events in the root population led us to remove 3 samples from analysis due to low quality. We ran FAUST on the remaining 66 samples, which consisted of 21 samples collected at cycle C01, before initiation of anti-PD-1 therapy; 16 at cycle C02; 18 at cycle C05; 1 at C08; and 10 at EOT. Of the 21 baseline samples, 1 was coded as non-evaluable “NE.” This sample was removed from downstream statistical analysis. Thirteen of the 20 subjects with baseline samples available responded to therapy (PR/CR), for an observed response rate of 65%.

FAUST was applied to the subset of CD45^+^ singlets with the experimental unit set to individual patient samples. After tuning, FAUST selected the markers CD11B, CD11C, CD14, CD16, CD19, CD20, CD33, CD3, CD56, and HLA-DR for the discovery and annotation of phenotypes. In the 20 baseline samples, FAUST phenotypes were tested for association with response to therapy (CR/PR) using a binomial GLMM with a subject-level random effect (the model is analogous to that specified in Equation 4.2). At the Bonferroni-adjusted 0.10 level, four phenotypes were significantly associated with response to therapy in baseline samples (supplemental experimental procedures A.2), including the phenotype discussed in “FAUST enables targeted hypothesis testing for pre-specified phenotypes.”

### CITN-07 phenotyping panel analysis

We ran FAUST on this dataset comprising of a total of 358 longitudinal samples from 35 subjects in two cohorts (cohort 1 with FLT-3 pre-treatment and cohort 2 without pre-treatment), with between 4 and 12 samples per subject over four cycles of therapy and at the end of the trial. Subjects were given FLT-3 ligand 7 days prior to the start of the first two of four treatment cycles. FLT-3 ligand was given to promote the expansion of myeloid and dendritic cell compartments to investigate whether expansion improved response to therapy. FAUST was configured to perform cell population discovery and annotation per sample in order to account for biological and technical heterogeneity. Debris, dead cells, and non-lymphocytes were excluded by pre-gating.

The CITN-07 phenotyping staining panel is described in supplemental experimental procedures A.27. FAUST tuning parameter settings are described in supplemental experimental procedures A.17. FAUST was applied to the pre-gated cells with the experimental unit set to individual patient samples. After tuning, FAUST selected the markers CD11C, CD123, CD14, CD16, CD19, CD3, CD4, CD56, CD8, and HLA-DR for the discovery and annotation of phenotypes. We tested each discovered cell population at the cohort-specific baseline (32 samples) for association with recurrence of disease (14 subjects had disease recurrence and 18 did not). We analyzed the baseline counts using a model similar to that of Equation 4.2. Here, the model was adjusted for subject-to-subject variability using a random effect, while cohort status, recurrence, and NYESO-1 staining of the tumor by immunohistochemistry (measured as positive, negative, or undetermined) were modeled as population effects.

### Krieg et al. FACS analysis

The Krieg et al.^9^ fluorescence-activated cell sorting (FACS) staining panel is described in supplemental experimental procedures A.28. FAUST tuning parameter settings are described in supplemental experimental procedures A.21. We used FAUST to process 31 baseline flow cytometry samples from responders and non-responders to therapy (16 responders and 15 non-responders). QC and review of the manual gating strategy led us to make manual adjustments to the “Lymphocytes” gate of 7 samples in this dataset. An example of this gate adjustment is shown in supplemental experimental procedures A.16.

FAUST was applied to live cells from the manual gating strategy used by Krieg et al.^9^ with the experimental unit set to individual samples. After tuning, FAUST selected 9 markers for discovery and annotation: CD11b, CD14, CD16, CD19, CD3, CD45RO, CD4, CD56, and HLA-DR. FAUST phenotypes were tested for association with responder status using a binomial GLMM with a subject-level random effect. The statistical model used here is identical to that of Equation 4.2, with $c_{i,\;k}$ now denoting the clusters in the FACS data and $n_{i}$ referring to the baseline FACS sample counts. At the Bonferroni-adjusted 0.10 level, 3 phenotypes were significantly associated with responder status (supplemental experimental procedures A.4), including the phenotype discussed in “FAUST enables targeted hypothesis testing for pre-specified phenotypes.”

### Subrahmanyam et al. CyTOF analysis

The markers used from the Subrahmanyam et al.^49^ CyTOF panel is described in supplemental experimental procedures A.29. FAUST tuning parameter settings are described in supplemental experimental procedures A.22. We used openCyto^74^ to reproduce the manual gating strategy reported by Subrahmanyam et al.^49^ to identify live intact singlets in each analyzed sample (supplemental experimental procedures A.15). We then used FAUST to process 64 pre-treatment, unstimulated CyTOF samples from responders and non-responders to ipilimumab (anti-CTLA-4, 10 responders, 14 non-responders) and pembrolizumab (anti-PD-1, 21 responders, 19 non-responders). FAUST was run through the boundary standardization phase at the individual-sample level on live intact singlets (as identified by openCyto). FAUST was used to determine standardized annotation boundaries for 10 markers: CD4, CD3, CD8, CD45RA, HLA-DR, CD28, PD-1, CD25, CD127, and CCR7. Once these boundaries were computed, cells with CD4^+^ or CD8^+^ phenotypes corresponding to significant phenotypes discovered in the MCC anti-PD-1 trial (see “CD8+ T cells from virus-positive subjects correlate with in-tumor measurements”) were targeted in each sample using the standardized boundaries. This produced counts for two cell populations. Counts from these two cell populations were taken from the 40 samples from subjects that went on to receive anti-PD-1 therapy, and tested for association with response to therapy. The model used here is identical to that of Equation 4.2, *mutatis mutandis*.

### PFDA multivariate model

We describe the multivariate PFDA model for the CD14^+^ CD16^−^ HLA-DR^+/bright^ cells; the T cell model is the same, modified only by the inclusion criteria. All FAUST phenotypes annotated as CD14^+^ CD16^−^ HLA-DR^+/bright^ CD3^−^ CD56^−^ CD19^−^ and included in the univariate analysis were selected in CITN-07 (the FLT3-ligand + therapeutic Vx trial), CITN-09 (the MCC anti-PD-1 trial), and the Krieg et al. melanoma anti-PD-1 trial FACS dataset.^9^ Let $k^{\text{∗}}$ denote the number of FAUST phenotypes within a given study. Let *n* denote the number of subjects at baseline, and $N=n\cdot k^{\text{∗}}$. For 1≤i≤N, $1\leq j\leq k^{\text{∗}}$ our statistical model is

$${\text{logit}}^{-1}\left(\mu_{i,\;j}\right)=\beta_{0}+\beta_{R}\cdot {\text{Responder}}_{i}$$

(Equation 4.3) $$+\sum_{j=1}^{k^{\text{∗}}}\left(\beta_{c,j}\cdot {\text{Cluster}}_{i,\;j}+\beta_{i,\;j}\cdot {\text{Cluster}}_{i,\;j}\cdot {\text{Responder}}_{i}\right)+\xi_{i}\text{,}$$

where Cluster_*i,j*_ is an indicator variable that is 1 when observation *i* is from cluster *j* and 0 otherwise, Responder_*i*_ is an indicator variable when observation *i* is taken from a responding subject, and $\eta_{i}∼N\left(0,\sigma_{i}^{2}\right)$ is an observation-level random effect. After estimating model coefficients $\beta_{i,\;j}$ in Equation 4.3, we test for differential abundance by testing t for positivity of linear combination of the coefficients:

$$H_{0}:\beta_{R}+\frac{1}{k^{\text{∗}}}\cdot \sum_{j=1}^{k^{\text{∗}}}\beta_{i,\;j}\leq 0\text{,}$$

$$H_{1}:\beta_{R}+\frac{1}{k^{\text{∗}}}\cdot \sum_{j=1}^{k^{\text{∗}}}\beta_{i,\;j}>0\text{.}$$

## Supplemental information

Document S1. Supplemental experimental procedures, Figures S1–S9, and Tables S1–S24

Document S2. Article plus supplemental information
