# Supplementary material for: New interpretable machine-learning method for single-cell data reveals correlates of clinical response to cancer immunotherapy
Source: Patterns (N Y). 2021 Oct 27;2(12):100372. doi: 10.1016/j.patter.2021.100372 (PMC8672150; doi:10.1016/j.patter.2021.100372)
Supplement: Document S1. Supplemental experimental procedures, Figures S1–S9, and Tables S1–S24 [file mmc1.pdf]

Patterns, Volume *2*

Supplemental information

New interpretable machine-learning method for single-cell data reveals correlates of clinical response to cancer immunotherapy

Evan Greene, Greg Finak, Leonard A. D'Amico, Nina Bhardwaj, Candice D. Church, Chihiro Morishima, Nirasha Ramchurren, Janis M. Taube, Paul T. Nghiem, Martin A. Cheever, Steven P. Fling, and Raphael Gottardo

Supplemental Experimental Procedures A. Supplementary materials

Supplemental Experimental Procedures A.1.

Table S1: Summary of CITN clinical trials

	CITN-07	CITN-09
ClinicalTrials.gov Identifier*	NCT02129075	NCT02267603
Study Type*	Interventional (Clinical Trial)	Interventional (Clinical Trial)
Intervention Model*	Parallel Assignment	Single Group Assignment
Masking*	None (Open Label)	None (Open Label)
Enrollment*	100 participants (Estimated, recorded 6/11/2019)	50 participants (Actual, recorded 6/11/2019)
Primary Purpose*	Treatment	Treatment
Actual Study Start Date*	April 9, 2014	November 25, 2014
Primary Completion Date*	February 2, 2020 (Estimated, recorded 6/11/2019)	February 6, 2018 (Actual, recorded 6/11/2019
Total number of longitudinal samples analyzed by FAUST	358	78 (T cell pane), 66 (Myeloid panel)
Total number of baseline samples (pretreatment)	32	27 (T cell panel), 20 (Myeloid panel)
Phase*	Phase 2	Phase 2
Intervention/Treatment*	Biological: DEC-205/NY-ESO-1 Fusion Protein CDX-1401 Other: Laboratory Biomarker Analysis Biological: Neoantigen-based Melanoma-Poly-ICLC Vaccine Other: Pharmacological Study Biological: Recombinant Flt3 Ligand	Biological: Pembrolizumab Other: Laboratory Biomarker Analysis
Condition/Disease*	Cutaneous Melanoma Mucosal Melanoma NY-ESO-1 Positive Tumor Cells Present Ocular Melanoma Stage IIB Cutaneous Melanoma AJCC v6 and v7 Stage IIC Cutaneous Melanoma AJCC v6 and v7 Stage III Cutaneous Melanoma AJCC v7 Stage IIIA Cutaneous Melanoma AJCC v7 Stage IIIB Cutaneous Melanoma AJCC v7 Stage IIIC Cutaneous Melanoma AJCC v7 Stage IV Cutaneous Melanoma AJCC v6 and v7	Recurrent Merkel Cell Carcinoma Stage III Merkel Cell Carcinoma AJCC v7 Stage IIIA Merkel Cell Carcinoma AJCC v7 Stage IIIB Merkel Cell Carcinoma AJCC v7 Stage IV Merkel Cell Carcinoma AJCC v7

This table contains data describing the CITN clinical trials CITN-07 and CITN-09. Data listed in all rows with * taken from https://clinicaltrials.gov on June 11, 2019.

Supplemental Experimental Procedures A.2.

Table S2: Baseline predictors MCC anti-PD-1 trial myeloid phenotyping panel

FAUST Phenotype	Bonferroni p-value
CD33 Bright CD16+ CD15+ CD14+ CD3- HLA-DR Bright CD20- CD19- CD11B+ CD56- CD11C+	0.0110
CD33 Bright CD16- CD15+ CD14+ CD3- HLA-DR Bright CD20- CD19- CD11B+ CD56- CD11C+	0.0138
CD33 Bright CD16- CD15- CD14+ CD3- HLA-DR Bright CD20- CD19- CD11B+ CD56- CD11C-	0.0289
CD33 Bright CD16- CD15- CD14+ CD3- HLA-DR Bright CD20- CD19- CD11B+ CD56- CD11C+	0.0309

All statistically significant (Bonferroni adjusted significance threshold of 10%) from the CITN-09 Myeloid panel.

Supplemental Experimental Procedures A.3.

Table S3: Baseline predictors FLT3-L + therapeutic Vx trial

FAUST Phenotype	Bonferroni p-value
CD8DimCD3-HLA DRDimCD19-CD14-CD11C-CD4-CD123-CD16+CD56-	0.0000
CD8-CD3-HLA DRDimCD19-CD14-CD11C-CD4-CD123-CD16+CD56-	0.0001
CD8DimCD3-HLA DR-CD19-CD14-CD11C-CD4-CD123-CD16+CD56-	0.0001
CD8-CD3-HLA DR-CD19-CD14-CD11C-CD4-CD123-CD16+CD56-	0.0003
CD8BrightCD3+HLA DRBrightCD19+CD14-CD11C-CD4DimCD123-CD16-CD56-	0.0047
CD8-CD3-HLA DRDimCD19-CD14+CD11C+CD4-CD123-CD16-CD56-	0.0073
CD8DimCD3-HLA DRDimCD19-CD14-CD11C-CD4-CD123-CD16+CD56+	0.0169
CD8DimCD3-HLA DRDimCD19-CD14+CD11C+CD4-CD123-CD16-CD56-	0.0179
CD8DimCD3-HLA DR-CD19-CD14-CD11C-CD4-CD123-CD16+CD56+	0.0319
CD8-CD3-HLA DRBrightCD19-CD14-CD11C-CD4-CD123-CD16-CD56-	0.0382
CD8BrightCD3+HLA DRBrightCD19-CD14-CD11C-CD4-CD123-CD16-CD56-	0.0438
CD8DimCD3-HLA DRDimCD19-CD14-CD11C+CD4-CD123-CD16+CD56-	0.0450
CD8-CD3-HLA DRBrightCD19-CD14+CD11C+CD4-CD123-CD16-CD56-	0.0460
CD8-CD3-HLA DRBrightCD19-CD14+CD11C+CD4-CD123-CD16+CD56-	0.0518
CD8-CD3-HLA DRDimCD19-CD14-CD11C-CD4-CD123-CD16+CD56+	0.0636
CD8DimCD3-HLA DRBrightCD19-CD14+CD11C+CD4-CD123-CD16-CD56-	0.0703
CD8BrightCD3+HLA DRDimCD19-CD14-CD11C-CD4DimCD123-CD16-CD56-	0.0755
CD8DimCD3-HLA DRBrightCD19+CD14-CD11C+CD4DimCD123-CD16-CD56-	0.0762
CD8-CD3-HLA DRDimCD19-CD14-CD11C-CD4-CD123-CD16-CD56-	0.0789
CD8-CD3-HLA DR-CD19-CD14-CD11C-CD4-CD123-CD16+CD56+	0.0883

All statistically significant (Bonferroni adjusted significance threshold of 10%) from the FLT3-L + therapeutic Vx trial.

Supplemental Experimental Procedures A.4.

Table S4: Predictors in Krieg et al. FACS analysis

FAUST Phenotype	Bonferroni p-value
CD3-CD4+HLA-DR+CD14+CD19-CD11b+CD16-CD56-CD45RO+	0.0000
CD3-CD4+HLA-DR+CD14+CD19-CD11b+CD16-CD56-CD45RO-	0.0016
CD3-CD4+HLA-DR+CD14+CD19-CD11b-CD16-CD56-CD45RO-	0.0683

All statistically significant (Bonferroni adjusted significance threshold of 10%) from the Krieg et al. FACS analysis

Supplemental Experimental Procedures A.5.

Figure S1: The temporal abundance of of manually gated PD-1+ CD8 T cells in the in MCC anti-PD-1 trial

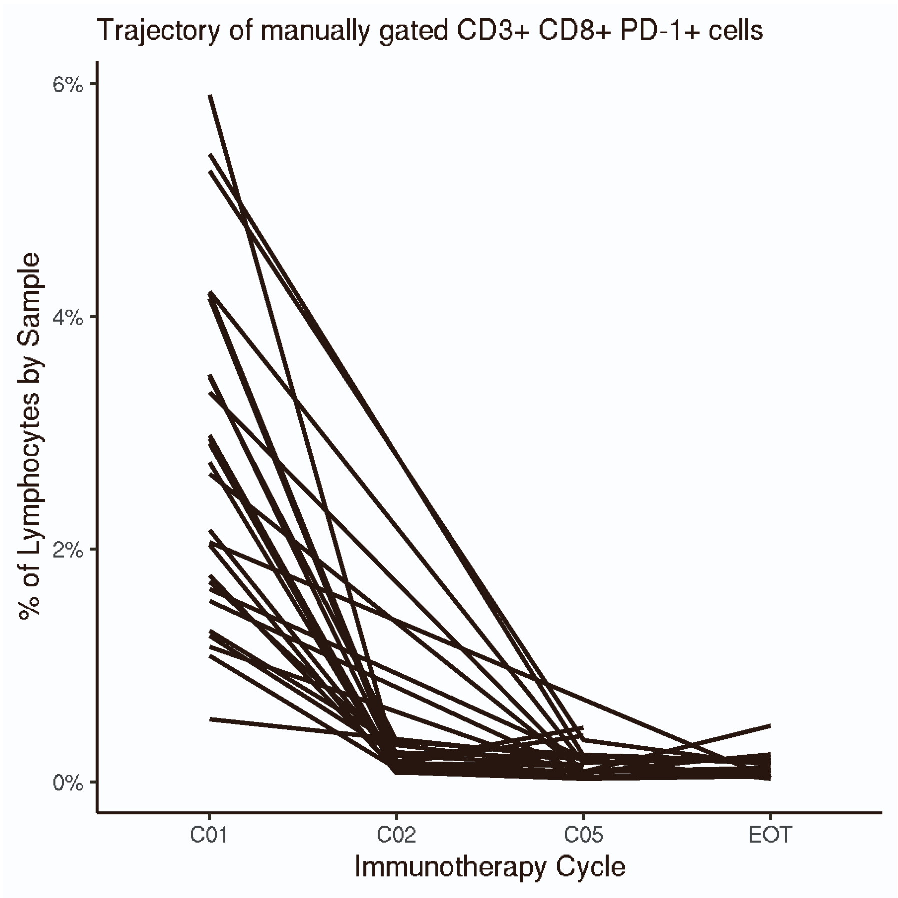

This figure displays the temporal abundance of manually gated CD3+ CD8+ PD-1+ cells across all collected time-points in the MCC anti-PD-1 trial discussed in section 2.4. Each line represents an individual subject.

Figure S2: Significant CD4 correlates discovered in CITN-09 MCC study

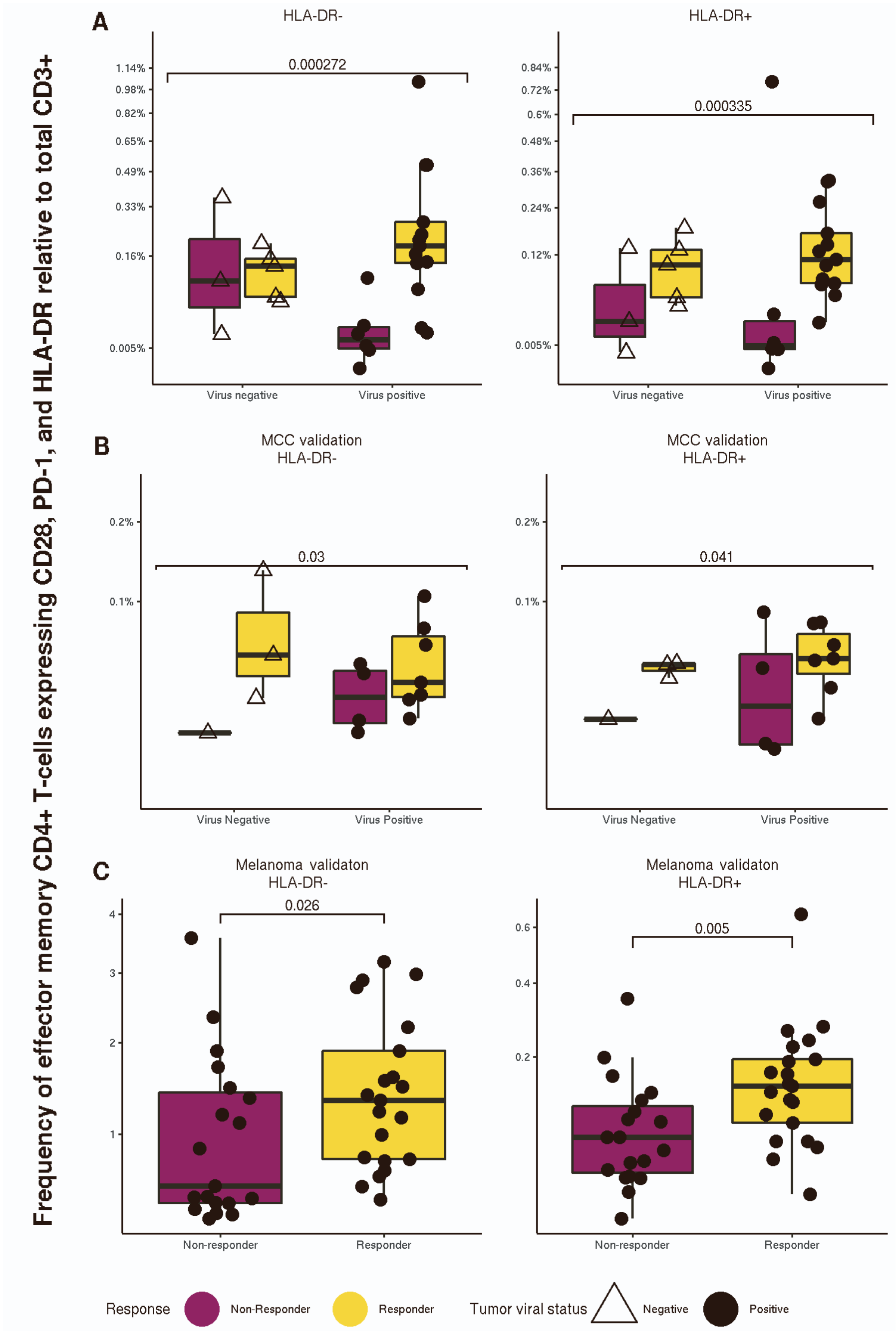

This figure displays data for the CD4 effector memory baseline correlates discovered in the MCC CITN-09 study. The full annotations produced by FAUST are CD4 Bright CD8- CD3+ CD45RA- HLA-DR- PD-1 Dim CD28+ CD127- CD25- CCR7- and CD4 Bright CD8- CD3+ CD45RA- HLA-DR+ PD1- Dim CD28+ CD127- CD25- CCR7-. Row A displays frequencies from the initial CITN-09 MCC analysis of fresh whole blood samples; Row B, frequencies from the CITN-09 cyropreserved PBMC dataset; Row C, the melanoma CyTOF dataset of Subrahmanyam et al.[59]

Figure S3: Examples of simulated data margins

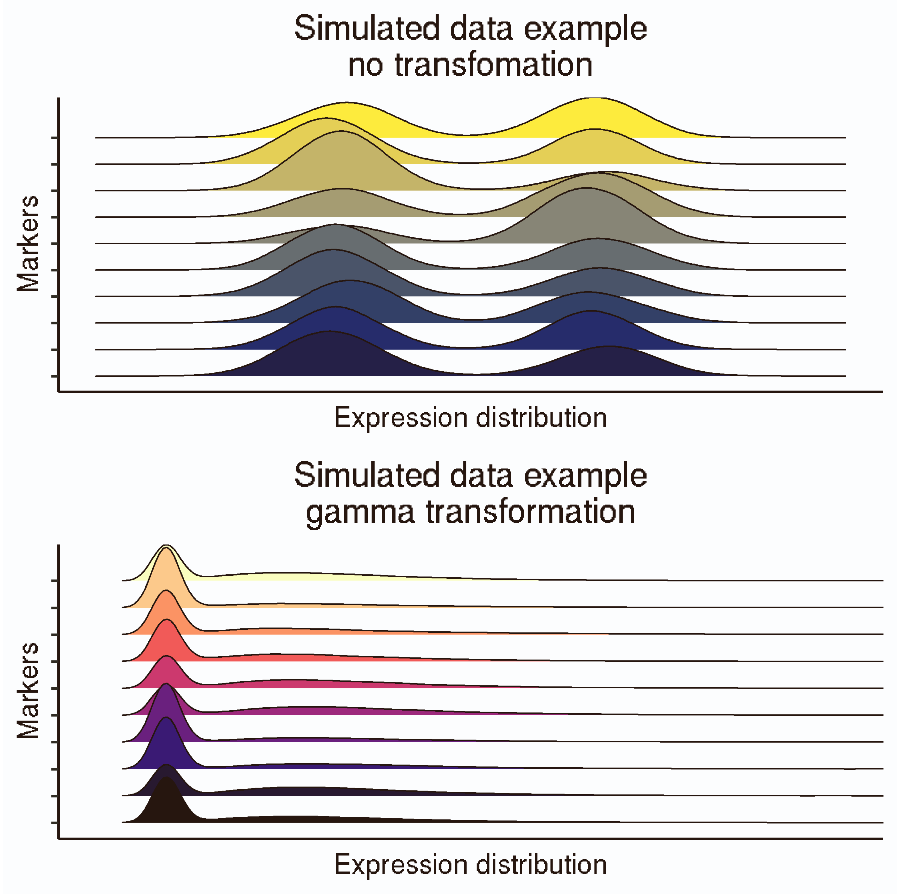

This figure displays the marginal distributions of a sample in a simulated dataset from the multivariate Gaussian simulation, as well as a simulated sample under the gamma transformation $\Gamma(1 + |x/4|)$.

Figure S4: Examples of tuning the marker boundary matrix

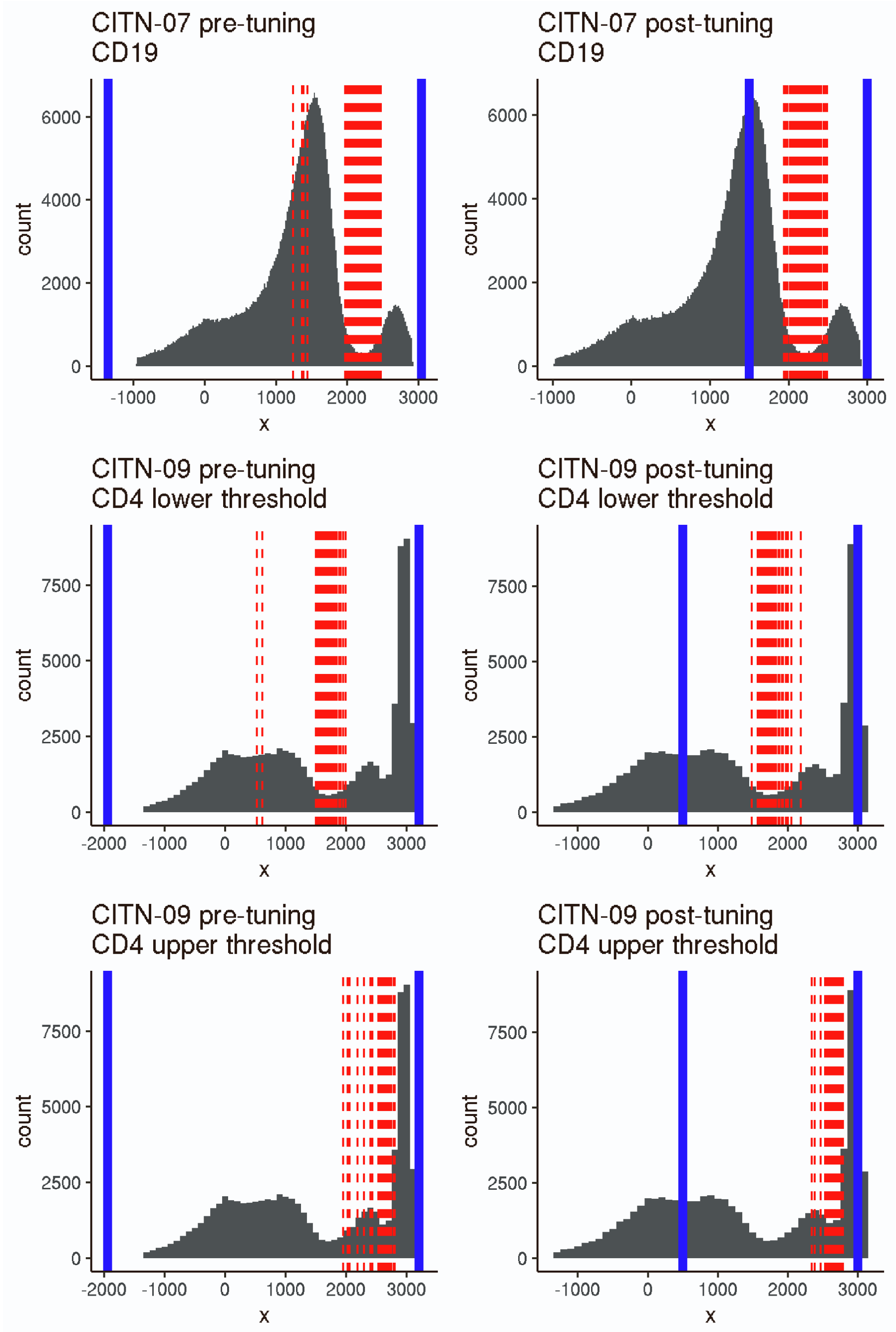

Examples from two datasets of a diagnostic plot showing an aggregate expression histogram for the stated marker, generated by sub-sampling (at most) 1000 observations per sample and concatenating. The distribution of annotation thresholds are displayed as red dashed lines, the marker boundary settings in blue. On the left are two example plots produced with the default marker boundary matrix. Based on these plots, we set the CD19 Low value to 1501 and the CD19 high value to 3000 in CITN-07, and the CD4 Low value to 500 and CD4 high value to 3000 in CITN-09. The result of this tuning is displayed on the right.

Supplemental Experimental Procedures A.9.

Figure S5: FAUST myeloid correlates across multiple datasets

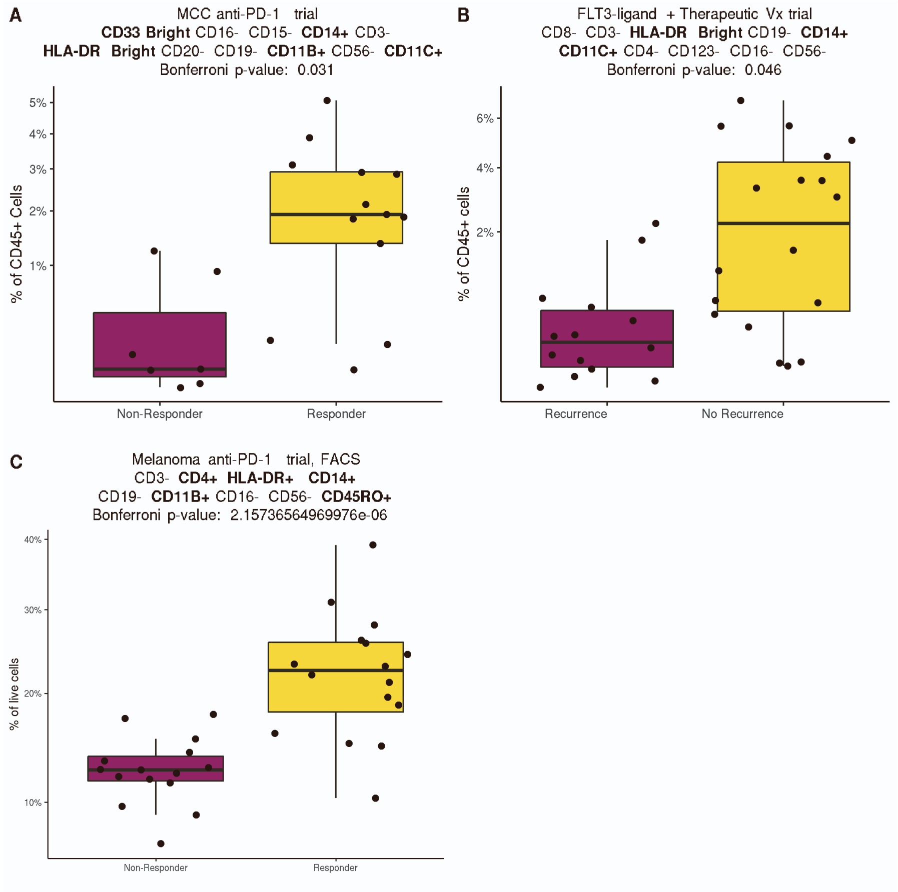

This figure displays significant myeloid correlates discovered through unbiased FAUST analysis of cytometry data generated in the CITN-09 trial, CITN-07 trial, and the melanoma trial described by Krieg et al.[36].

Supplemental Experimental Procedures A.10.

Figure S6: Number of cluster ARI simulation results on the FAUST subset annotated with down-selected phenotypes

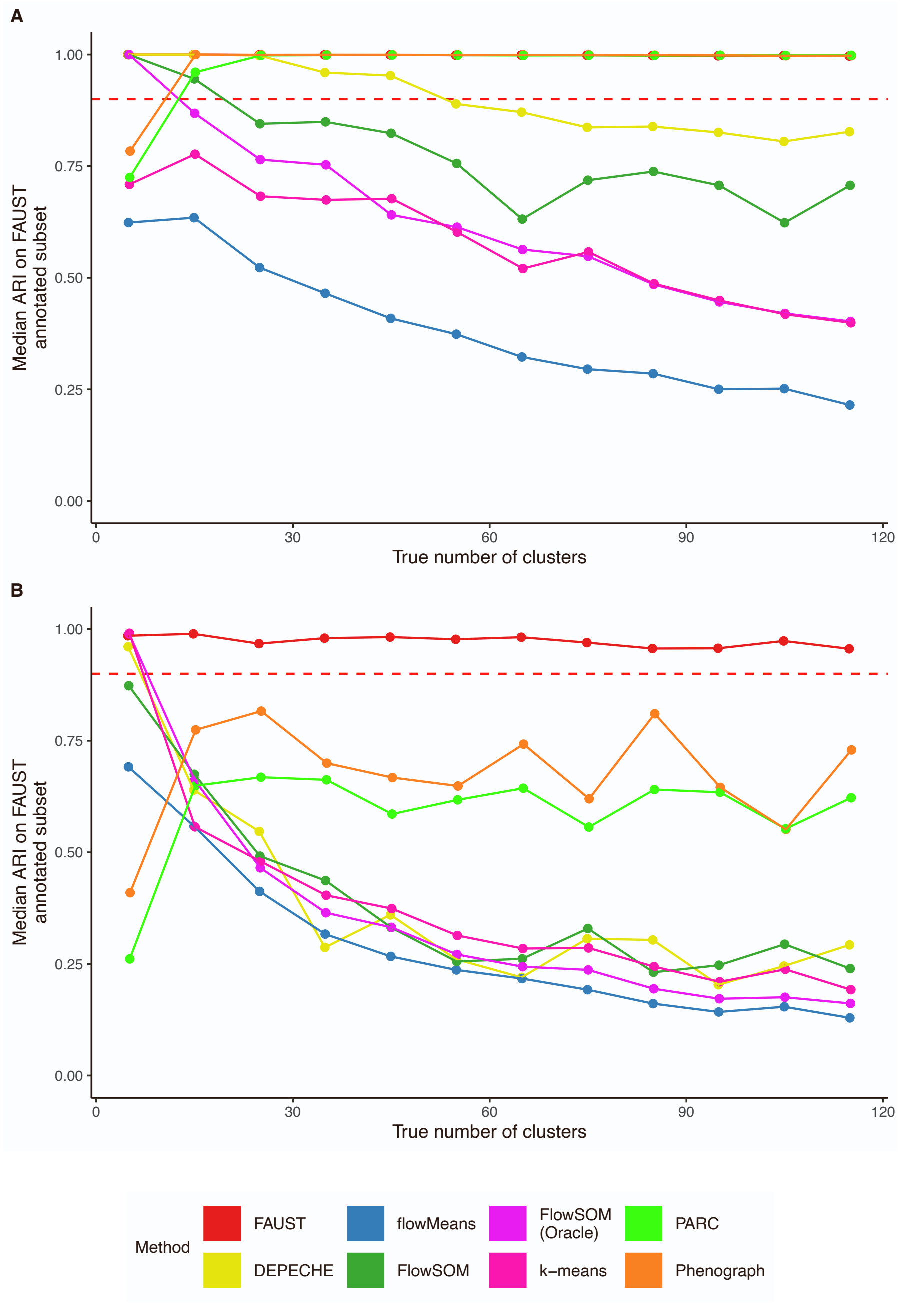

This figure displays method performance on the subset of observations in each sample annotated by FAUST's down-selected phenotypes for all methods in A) the multivariate Gaussian number of clusters simulation B) the $\Gamma(1 + |x/4|)$ transformed number of clusters simulation.

Figure S7: Multi-threaded implementation accelerates FAUST run times

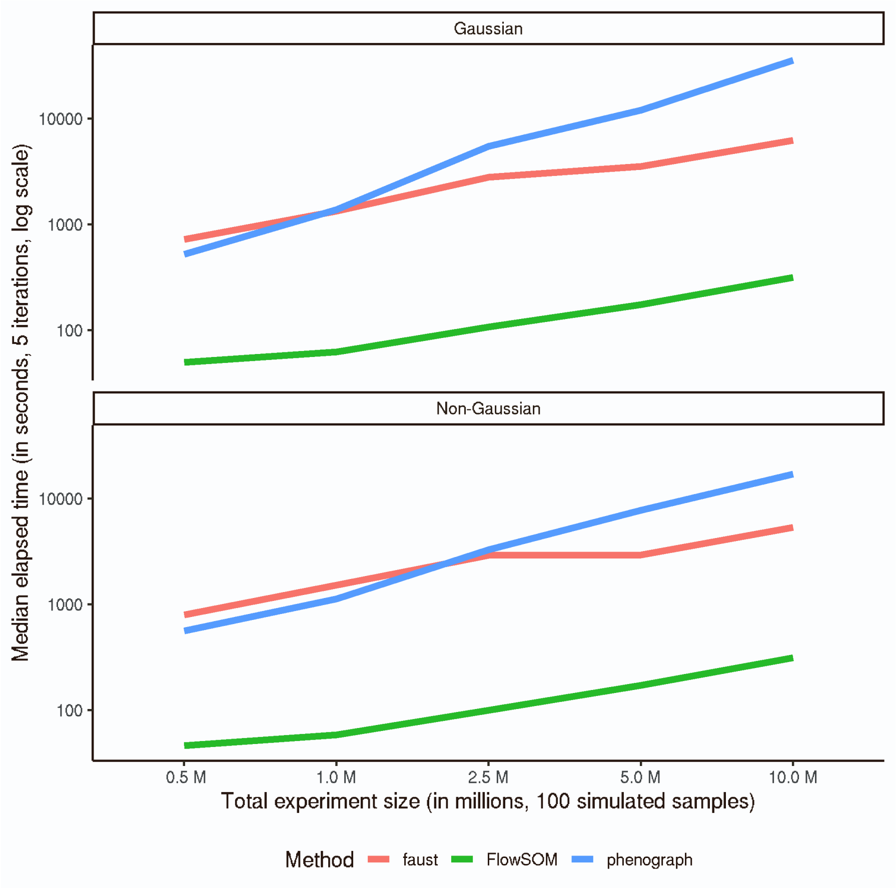

Here we report run time data obtained from adapting the simulation study described in Experimental Procedures section 4.10 to generate simulated experiments of 100 samples ranging from 0.5 million to 10 million total observations. This produced the simulated sample sizes ranging between 5000 and 100,000 observations. We applied FAUST with 10 threads, phenograph[12,39], and FlowSOM[65,66] to these simulated datasets. We observed FlowSOM was the fastest running method, that FAUST and phenograph had similar run times on the smaller simulated experiments, and that as the total experiment size increased the phenograph implementation we applied had longer median run time than FAUST with 10 threads.

This figure shows the elapsed run time measurements of FAUST with 10 threads, phenograph, and FlowSOM applied to simulated experiments with 100 simulated samples from A) the multivariate Gaussian number of clusters simulation B) the $\Gamma(1 + |x/4|)$ transformed number of clusters simulation described in Experimental Procedures 4.10.

Supplemental Experimental Procedures A.12.

Table S5: Other methods applied to CITN-09

	Method	Adjustment	Cluster 1	Cluster 2	Cluster 3	Cluster 4	Cluster 5
1	FlowSOM, 10x10 grid	Bonferroni	1	1	1	1	1
2	FlowSOM, 20x20 grid	Bonferroni	0.42	0.813	0.983	1	1
3	kmeans, k=100	Bonferroni	1	1	1	1	1
4	kmeans, k=400	Bonferroni	0.368	0.428	0.875	1	1
5	PARC	Bonferroni	0.776	0.848	1	1	1
6	Phenograph	Bonferroni	1	1	1	1	1
7	FlowSOM, 10x10 grid	FDR	0.649	0.649	0.649	0.649	0.649
8	FlowSOM, 20x20 grid	FDR	0.328	0.328	0.328	0.356	0.356
9	kmeans, k=100	FDR	0.625	0.625	0.625	0.633	0.661
10	kmeans, k=400	FDR	0.214	0.214	0.292	0.372	0.372
11	PARC	FDR	0.291	0.291	0.291	0.291	0.291
12	Phenograph	FDR	0.335	0.335	0.335	0.335	0.335

Results of applying the clustering methods FlowSOM, kmeans, PARC, and Phenograph to flow cytometry data stained to investigate T cell activity from the MCC anti-PD-1 trial.

Supplemental Experimental Procedures A.13.

Table S6: Additional sub-populations associated with with clinical outcome at baseline in CITN-09

Phenotype	FDR-adjusted p-value
CD4 Bright CD8- CD3+ CD45RA- HLADR- PD1 Dim CD28+ CD127- CD25- CCR7-	0.002
CD4- CD8+ CD3+ CD45RA- HLADR+ PD1 Dim CD28+ CD127- CD25- CCR7-	0.004
CD4- CD8+ CD3+ CD45RA- HLADR+ PD1 Bright CD28+ CD127- CD25- CCR7-	0.015
CD4 Bright CD8- CD3+ CD45RA- HLADR+ PD1 Dim CD28+ CD127- CD25- CCR7-	0.015
CD4 Bright CD8- CD3+ CD45RA- HLADR- PD1 Dim CD28+ CD127- CD25- CCR7+	0.026
CD4- CD8+ CD3+ CD45RA- HLADR- PD1 Dim CD28+ CD127- CD25- CCR7-	0.047
CD4- CD8+ CD3+ CD45RA+ HLADR- PD1 Bright CD28- CD127- CD25- CCR7-	0.052
CD4 Bright CD8- CD3+ CD45RA+ HLADR- PD1 Dim CD28- CD127- CD25- CCR7-	0.087
CD4 Bright CD8- CD3+ CD45RA+ HLADR- PD1 Dim CD28+ CD127- CD25- CCR7+	0.109
CD4- CD8+ CD3+ CD45RA- HLADR- PD1 Bright CD28+ CD127- CD25- CCR7-	0.132
CD4- CD8+ CD3+ CD45RA+ HLADR- PD1 Dim CD28- CD127- CD25- CCR7-	0.132
CD4- CD8+ CD3+ CD45RA+ HLADR- PD1 Dim CD28+ CD127- CD25- CCR7-	0.132
CD4- CD8+ CD3+ CD45RA+ HLADR- PD1 Dim CD28+ CD127+ CD25+ CCR7+	0.132
CD4 Bright CD8- CD3+ CD45RA- HLADR- PD1- CD28+ CD127- CD25- CCR7+	0.132
CD4 Bright CD8- CD3+ CD45RA- HLADR- PD1 Dim CD28- CD127- CD25- CCR7-	0.132
CD4 Bright CD8- CD3+ CD45RA- HLADR- PD1 Dim CD28+ CD127- CD25+ CCR7-	0.132
CD4 Bright CD8- CD3+ CD45RA- HLADR- PD1 Dim CD28+ CD127+ CD25- CCR7-	0.132
CD4 Bright CD8- CD3+ CD45RA- HLADR- PD1 Dim CD28+ CD127+ CD25- CCR7+	0.132
CD4 Bright CD8- CD3+ CD45RA- HLADR- PD1 Bright CD28+ CD127- CD25- CCR7-	0.132

CD4 Bright CD8- CD3+ CD45RA- HLADR- PD1 Bright CD28+ CD127- CD25- CCR7+	0.132
CD4 Bright CD8- CD3+ CD45RA+ HLADR- PD1- CD28+ CD127- CD25- CCR7+	0.132
CD4- CD8- CD3+ CD45RA+ HLADR- PD1 Dim CD28+ CD127- CD25- CCR7-	0.136
CD4- CD8+ CD3+ CD45RA- HLADR+ PD1- CD28+ CD127- CD25- CCR7-	0.136
CD4 Dim CD8+ CD3+ CD45RA- HLADR- PD1 Dim CD28+ CD127+ CD25- CCR7-	0.137
CD4- CD8+ CD3+ CD45RA- HLADR- PD1 Dim CD28+ CD127+ CD25- CCR7-	0.149
CD4- CD8+ CD3+ CD45RA+ HLADR- PD1 Dim CD28- CD127- CD25+ CCR7-	0.149
CD4 Bright CD8- CD3+ CD45RA- HLADR- PD1 Dim CD28- CD127+ CD25- CCR7-	0.149
CD4 Bright CD8- CD3+ CD45RA+ HLADR- PD1- CD28- CD127- CD25- CCR7-	0.149
CD4 Bright CD8- CD3+ CD45RA+ HLADR- PD1- CD28+ CD127+ CD25- CCR7+	0.157
CD4- CD8+ CD3+ CD45RA- HLADR+ PD1 Dim CD28- CD127- CD25- CCR7-	0.162
CD4- CD8- CD3+ CD45RA+ HLADR- PD1 Dim CD28+ CD127- CD25- CCR7+	0.179
CD4- CD8+ CD3+ CD45RA- HLADR- PD1- CD28+ CD127- CD25- CCR7-	0.179
CD4- CD8+ CD3+ CD45RA- HLADR- PD1 Dim CD28- CD127- CD25- CCR7-	0.179
CD4- CD8+ CD3+ CD45RA- HLADR+ PD1 Dim CD28+ CD127- CD25- CCR7+	0.179
CD4- CD8+ CD3+ CD45RA- HLADR+ PD1 Bright CD28+ CD127- CD25- CCR7+	0.179
CD4- CD8+ CD3+ CD45RA+ HLADR- PD1- CD28+ CD127+ CD25+ CCR7+	0.179
CD4 Bright CD8- CD3+ CD45RA- HLADR- PD1- CD28- CD127- CD25- CCR7-	0.179
CD4 Bright CD8- CD3+ CD45RA- HLADR- PD1- CD28+ CD127+ CD25- CCR7+	0.179
CD4 Bright CD8- CD3+ CD45RA- HLADR+ PD1 Dim CD28+ CD127+ CD25- CCR7-	0.179
CD4 Bright CD8- CD3+ CD45RA- HLADR+ PD1 Bright CD28+ CD127- CD25- CCR7-	0.179
CD4 Bright CD8- CD3+ CD45RA+ HLADR- PD1 Dim CD28- CD127- CD25+ CCR7-	0.179
CD4 Bright CD8- CD3+ CD45RA+ HLADR- PD1 Dim CD28+ CD127+ CD25- CCR7+	0.179

CD4 Bright CD8- CD3+ CD45RA- HLADR+ PD1 Dim CD28+ CD127- CD25+ CCR7-	0.194
CD4 Bright CD8- CD3+ CD45RA+ HLADR- PD1- CD28- CD127- CD25+ CCR7+	0.197

This table displays all FAUST Phenotypes associated with outcome in the CITN-09 T cell data at the FDR-adjusted 20% level.

Supplemental Experimental Procedures A.14.

Table S7: Effect Sizes and Confidence Intervals in CITN-09 T cell panel

Population	Effect Size (Log odds)	Lower 2.5%	Upper 97.5%
CD4 Bright CD8- CD3+ CD45RA- HLA-DR- PD-1 Dim CD28+ CD127- CD25- CCR7-	2.183	1.210	3.224
CD4- CD8+ CD3+ CD45RA- HLA-DR+ PD-1 Dim CD28+ CD127- CD25- CCR7-	1.912	1.000	2.914
CD4- CD8+ CD3+ CD45RA- HLA-DR+ PD-1 Bright CD28+ CD127- CD25- CCR7-	1.978	0.891	3.116
CD4 Bright CD8- CD3+ CD45RA- HLA-DR+ PD-1 Dim CD28+ CD127- CD25- CCR7-	1.940	0.866	3.081

This table contains effect sizes and confidence intervals for the four phenotypes detected in the FAUST CITN-09 T cell analysis at the Bonferroni-adjusted 0.10 level.

Figure S8: openCyto Gating Strategy replication

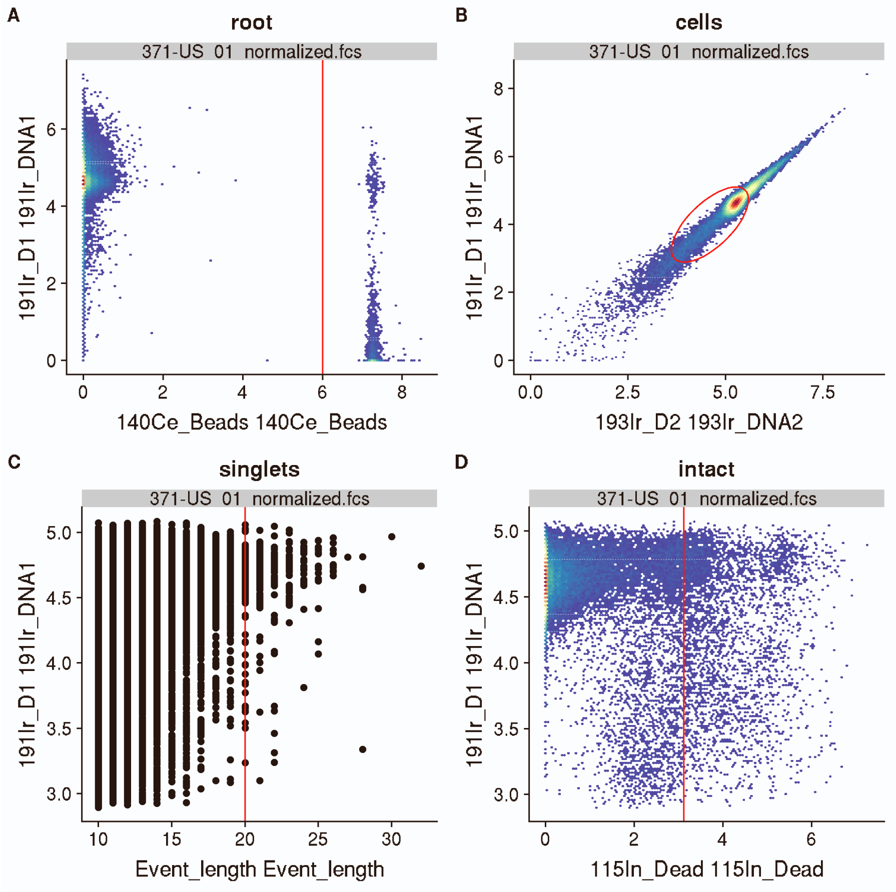

This supplementary figure shows an example of the openCyto[17] replication of the gating strategy described in Figure 1 of[59]. In the displayed sample, live intact singlets are identified.

Supplemental Experimental Procedures A.16.

Figure S9: Gating strategy modification examples

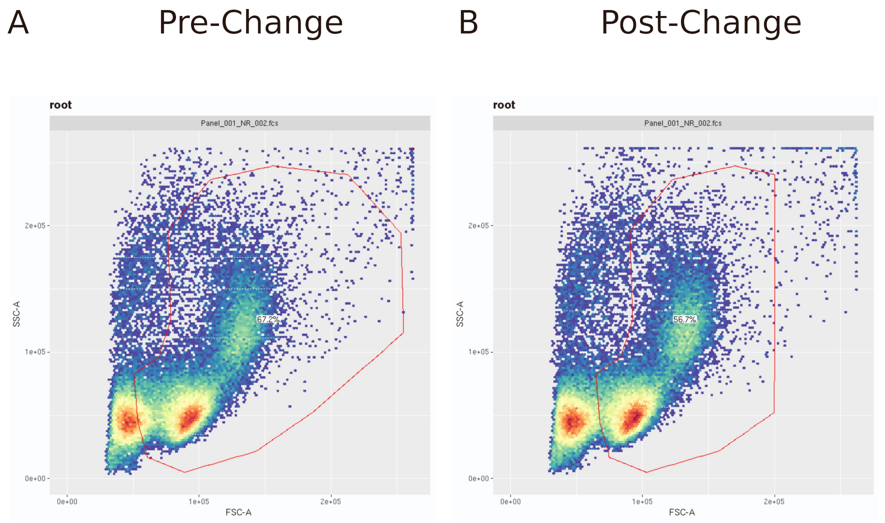

An example of modification to the manual gating strategy of the Krieg et al. FACS data. Panel A shows the initial manual gating strategy for the Lymphocytes of a sample. Panel B shows the same sample with the modified gate.

Supplemental Experimental Procedures A.17.

Table S8: CITN-07 Phenotyping panel FAUST parameter settings

	Low	High
CD123	1500.00	3000.00
CD4	100.00	2500.00
CD14	1.00	3000.00
CD11C	1.00	3000.00
CD56	1.00	3000.00
CD8	1.00	3000.00
CD16	1000.00	3000.00
CD3	1.00	3000.00
CD122	1000.00	3000.00
CD19	1501.00	3000.00
HLA DR	1.00	3500.00

This table displays the marker boundary matrix for CITN-07. Specific values were set by inspecting histograms of the individual markers across individual samples.The experimental unit was set to the individual sample. The imputation hierarchy was set to sample time point (with all follow-up appointments treated as a single time-point). The supervised list was used to encourage CD4 to have two annotation boundaries estimated. The phenotype occurrence number was set to 50. The starting cell population was "45+" cells in the manual gating strategy.

Supplemental Experimental Procedures A.18.

Table S9: CITN-09 T cell panel FAUST parameter settings

	Low	High
CD278 ICOS	-500.00	3000.00
CD3	-500.00	3000.00
CD127	-500.00	3000.00
CD197 CCR7	500.00	2250.00
CD279 PD1	-250.00	3500.00
CD8	500.00	3000.00
CD4	500.00	3000.00
CD28	-500.00	3000.00
CD25	500.00	2250.00
HLA DR	-500.00	3000.00
CD45RA	1000.00	3000.00

This table displays the marker boundary matrix for the CITN-09 T cell panel. Non-zero values were set by inspecting histograms of the individual samples.The supervised list was used to encourage PD-1 to have two annotation boundaries estimated if supported by the data. The phenotype occurrence number was set to 7. The experimental unit was set to the individual sample. The imputation hierarchy was to visit. The starting cell population was ”L” cells (live lymphocytes) in the manual gating strategy.

Supplemental Experimental Procedures A.19.

Table S10: CITN-09 cryopreserved PBMC analysis FAUST parameter settings

	Low	High
CD3	1200.00	2690.34
CD127	750.00	1750.00
CD197 CCR7	1500.00	2250.00
CD279 PD1	1400.00	2700.00
CD8	1800.00	2800.00
CD4	-698.51	3007.58
CD28	500.00	1750.00
CD25	-500.00	2278.36
HLA DR	-798.62	3504.31
CD45RA	1500.00	2750.00

This table displays the marker boundary matrix for the CITN-09 cryopreserved PBMC analysis. Non-zero values were set by inspecting histograms of the individual samples. The experimental unit was set to the individual sample. The selection quantile was set to 1.0. The depth score threshold was set to 0.01. The starting cell population was "L" cells (live lymphocytes) in the manual gating strategy.

Supplemental Experimental Procedures A.20.

Table S11: CITN-09 Myeloid panel FAUST parameter settings

	IH01_Low	IH01_High	IH02_Low	IH02_High	IH03_Low	IH03_High	IH04_Low	IH04_High
CD11B	1000	Inf	-1500	Inf	-1500	Inf	0	Inf
CD20	1000	Inf	-1000	Inf	0	Inf	-2500	Inf
CD14	1000	Inf	1000	Inf	500	Inf	1000	Inf
CD11C	1000	Inf	-5000	Inf	-5000	Inf	1000	Inf
CD56	2000	Inf	-2000	Inf	-2000	Inf	2300	Inf
CD33	-1000	2100	2200	Inf	0	Inf	-20000	Inf
CD16	-Inf	Inf	1000	Inf	1000	Inf	1000	Inf
CD3	1000	Inf	-3000	Inf	-5000	Inf	2000	Inf
CD15	1000	Inf	1000	Inf	0	Inf	0	Inf
CD19	1750	Inf	-1000	Inf	-1000	Inf	-1000	Inf
HLA DR	-1000	3750	1000	3750	-500	3750	-2500	3750

This table displays the marker boundary matrices for the CITN-09 Myeloid panel FAUST analysis.The experimental unit was set to the individual sample. The imputation hierarchy was set to four distinct values, with the marker boundary matrices of each value reported above. The supervised list was used to encourage both CD33 and HLA-DR to have two annotation boundaries estimated, and CD15 one. The phenotype occurrence number was set to 9. The starting cell population was "45+" cells in the manual gating strategy.

Supplemental Experimental Procedures A.21.

Table S12: Krieg et al. FACS FAUST parameter settings

	CD3	CD4	CD11b	CD33	HLA-DR	CD56	CD45RO	CD11c	CD16	CD14	CD19
Low	-Inf	-20.00	-Inf	-Inf	-Inf	-Inf	-Inf	-Inf	85.00	-Inf	-Inf
High	Inf	Inf	Inf	Inf	Inf	Inf	Inf	Inf	Inf	Inf	Inf

This table displays the marker boundary matrix for the FAUST analysis of the Krieg et al. FACS dataset. The experimental unit was set to the individual sample. The selection quantile was set to 0. The depth score threshold was set to 0.01. The supervised list was not used. The phenotype occurrence number was set to 10. The starting cell population was "life" cells in the manual gating strategy.

Supplemental Experimental Procedures A.22.

Table S13: Subrahmanyam et al. CyTOF FAUST parameter settings

	Low	High
145Nd_CD4	0.75	3.75
146Nd_CD8	1.00	4.00
141Pr_CD25	0.25	1.50
153Eu_CD45RA	1.00	5.00
165Ho_CD127	0.00	5.32
169Tm_CCR7	0.00	2.75
154Sm_CD3	0.00	6.23
157Gd_HLA-DR	2.00	6.25
170Er_PD-1	0.25	2.00
155Gd_CD28	0.25	4.54

This table displays the marker boundary matrix for the FAUST analysis of the Subrahmanyam et al. CyTOF dataset.The experimental unit was set to the individual sample. The selection quantile was set to 1.0. The depth score threshold was set to 0.01. The supervised list was not used. The phenotype occurance number was not used, since FAUST was terminated after the annotation boundaries were standardized across samples. The starting cell population was ”live” cells in the manual gating strategy recreated using openCyto.

Supplemental Experimental Procedures A.23.

Table S14: flowCAP IV FAUST parameters training set

	Low	High
CD4	0.50	3.41
CD27	0.50	2.75
CD8	0.50	2.50
CD57	0.50	3.58
CD45RO	0.50	2.75
CD107A	0.50	2.82
CD154	0.50	2.78
CCR7	0.50	2.53
IFNG	0.50	2.60
TNFA	0.50	1.50
IL2	0.50	2.55

Table S15: This table displays the marker boundary matrix used in the discovery analysis on the flowCAP IV training set. The experimental unit for the training set analysis was set to the individual subject, so that each subject's stimulated and unstimulated samples were concatenated. The selection quantile for the training set analysis was set to 0.75. The depth score threshold for the training set analysis was set to 0.005. The starting cell population for the training set analysis was the live T cells identified by the floReMi preprocessing script.

Supplemental Experimental Procedures A.24.

Table S16: flowCAP IV FAUST parameters test set

	Low	High
CD4	0.50	3.41
CD8	0.50	2.50
CD27	0.50	2.75
CD57	0.50	3.58
CD45RO	0.50	2.75
CD154	0.50	2.78
CCR7	0.50	2.53

This table displays the marker boundary matrix used in the FAUST analysis of the flowCAP IV test set. The subset of markers displayed were selected on the basis of the training set analysis. The selection quantile for the test set analysis was set to 1.0. The depth score threshold for the test set analysis was set to $1e - 6$. The experimental unit for the test set analysis was set to the individual subject, so that each subject's stimulated and unstimulated samples were concatenated. The starting cell population for the test set analysis was the live T cells identified by the floReMi preprocessing script.

Supplemental Experimental Procedures A.25.

Table S17: CITN-09 T cell Staining Panel

	name	desc
$P1	FSC-A	
$P2	FSC-H	
$P3	SSC-A	
$P4	SSC-H	
$P5	<PE-A>	CD278 ICOS
$P6	<FITC-A>	CD3
$P7	<BV 421-A>	CD127
$P8	<Alexa Fluor 700-A>	CD197 CCR7
$P9	<PE-Cy7-A>	CD279 PD-1
$P10	<PerCP-Cy5-5-A>	CD8
$P11	<APC-Cy7-A>	CD4
$P12	<ECD-A>	CD28
$P13	<APC-A>	CD25
$P14	PE-Cy5-A	
$P15	<AmCyan-A>	CD45
$P16	<BV 605-A>	HLA DR
$P17	<BV 650-A>	CD45RA
$P18	Time	

This table displays the channels and markers in the CITN-09 T cell staining panel.

Supplemental Experimental Procedures A.26.

Table S18: CITN-09 Myeloid Staining Panel

	name	desc
$P1	FSC-A	
$P2	FSC-H	
$P3	SSC-A	
$P4	SSC-H	
$P5	<PE-A>	CD11B
$P6	<FITC-A>	CD20
$P7	<BV 421-A>	CD14
$P8	<Alexa Fluor 700-A>	CD11C
$P9	<PE-Cy7-A>	CD56
$P10	<PerCP-Cy5-5-A>	CD33
$P11	<APC-Cy7-A>	CD16
$P12	<ECD-A>	CD3
$P13	<APC-A>	CD15
$P14	<PE-Cy5-A>	CD19
$P15	<AmCyan-A>	CD45
$P16	<BV 605-A>	HLA DR
$P17	BV 650-A	
$P18	Time	

This table displays the channels and markers in the CITN-09 Myeloid staining panel.

Supplemental Experimental Procedures A.27.

Table S19: CITN-07 Phenotyping Staining Panel

	name	desc
$P1	FSC-A	
$P2	FSC-H	
$P3	SSC-A	
$P4	SSC-H	
$P5	<PE-A>	CD123
$P6	<FITC-A>	CD4
$P7	<BV 421-A>	CD14
$P8	<Alexa Fluor 700-A>	CD11C
$P9	<PE-Cy7-A>	CD56
$P10	<PerCP-Cy5-5-A>	CD8
$P11	<APC-Cy7-A>	CD16
$P12	<ECD-A>	CD3
$P13	<APC-A>	CD122
$P14	<PE-Cy5-A>	CD19
$P15	<AmCyan-A>	CD45
$P16	<BV 605-A>	HLA DR
$P17	BV 650-A	
$P18	Time	

This table displays the channels and markers in the CITN-07 phenotyping staining panel.

Table S20: Krieg et al. FACS Panel

	name	desc
$P1	FSC-A	
$P2	FSC-H	
$P3	FSC-W	
$P4	SSC-A	
$P5	SSC-H	
$P6	SSC-W	
$P7	Comp-Brilliant Violet 785-A	CD3
$P8	Comp-Brilliant Violet 711-A	CD4
$P9	Comp-Brilliant Violet 421-A	CD11b
$P10	Comp-PerCP-Cy5-5-A	CD33
$P11	Comp-FITC-A	HLA-DR
$P12	Comp-PE-Cy7-A	CD56
$P13	Comp-PE-Texas Red-A	CD45RO
$P14	Comp-APC-Cy7-A	NIR
$P15	Comp-Alexa Fluor 700-A	CD11c
$P16	Comp-APC-A	CD16
$P17	Comp-PE-A	CD14
$P18	Comp-Brilliant Violet 605-A	CD19
$P19	Time	

This table displays the channels and markers in the FACS staining panel of Krieg et al.

Supplemental Experimental Procedures A.29.

Table S21: Subrahmanyam et al. CyTOF Panel

	name	desc
$P1	Time	Time
$P2	Event_length	Event_length
$P3	115In_Dead	115In_Dead
$P4	140Ce_Beads	140Ce_Beads
$P5	141Pr_CD25	141Pr_CD25
$P6	142Nd_CD19	142Nd_CD19
$P7	143Nd_IL-10	143Nd_IL-10
$P8	144Nd_IL-4	144Nd_IL-4
$P9	145Nd_CD4	145Nd_CD4
$P10	146Nd_CD8	146Nd_CD8
$P11	147Sm_CD20	147Sm_CD20
$P12	148Nd_CD57	148Nd_CD57
$P13	149Sm_CTLA-4	149Sm_CTLA-4
$P14	150Nd_MIP-1b	150Nd_MIP-1b
$P15	151Eu_CD107a	151Eu_CD107a
$P16	152Sm_TNFa	152Sm_TNFa
$P17	153Eu_CD45RA	153Eu_CD45RA
$P18	154Sm_CD3	154Sm_CD3
$P19	155Gd_CD28	155Gd_CD28
$P20	156Gd_CD38	156Gd_CD38
$P21	157Gd_HLA-DR	157Gd_HLA-DR
$P22	158Gd_CD33	158Gd_CD33
$P23	159Tb_GM-CSF	159Tb_GM-CSF
$P24	160Gd_CD14	160Gd_CD14
$P25	161Dy_IFNg	161Dy_IFNg
$P26	162Dy_CD69	162Dy_CD69
$P27	163Dy_TCRgd	163Dy_TCRgd
$P28	164Dy_IL-17	164Dy_IL-17
$P29	165Ho_CD127	165Ho_CD127
$P30	166Er_IL-2	166Er_IL-2
$P31	167Er_CD27	167Er_CD27
$P32	168Er_CD154	168Er_CD154
$P33	169Tm_CCR7	169Tm_CCR7
$P34	170Er_PD-1	170Er_PD-1
$P35	171Yb_GranzymeB	171Yb_GranzymeB
$P36	172Yb_PD-L2	172Yb_PD-L2
$P37	173Yb_Perforin	173Yb_Perforin
$P38	174Yb_CD16	174Yb_CD16
$P39	175Lu_PD-L1	175Lu_PD-L1
$P40	176Yb_CD56	176Yb_CD56
$P41	191Ir_DNA1	191Ir_DNA1
$P42	193Ir_DNA2	193Ir_DNA2

This table displays the names and description of markers in the Subrahmanyam et al. CyTOF Panel

Supplemental Experimental Procedures A.30.

Table S22: CITN-09 T cell Manual Gating Strategy

1	root
2	/Singlets
3	/Singlets/45
4	/Singlets/45/Lymphocytes
5	/Singlets/45/Lymphocytes/CD3
6	/Singlets/45/Lymphocytes/CD3/4
7	/Singlets/45/Lymphocytes/CD3/4/CD25+
8	/Singlets/45/Lymphocytes/CD3/4/CD25+CD45RA+CCR7+
9	/Singlets/45/Lymphocytes/CD3/4/CD25+CD45RA+CCR7-
10	/Singlets/45/Lymphocytes/CD3/4/CD25+CD45RA-CCR7+
11	/Singlets/45/Lymphocytes/CD3/4/CD25+CD45RA-CCR7-
12	/Singlets/45/Lymphocytes/CD3/4/CD25-CD45RA+CCR7+
13	/Singlets/45/Lymphocytes/CD3/4/CD25-CD45RA+CCR7-
14	/Singlets/45/Lymphocytes/CD3/4/CD25-CD45RA-CCR7+
15	/Singlets/45/Lymphocytes/CD3/4/CD25-CD45RA-CCR7-
16	/Singlets/45/Lymphocytes/CD3/4/CD28+
17	/Singlets/45/Lymphocytes/CD3/4/CD28+CD45RA+CCR7+
18	/Singlets/45/Lymphocytes/CD3/4/CD28+CD45RA+CCR7-
19	/Singlets/45/Lymphocytes/CD3/4/CD28+CD45RA-CCR7+
20	/Singlets/45/Lymphocytes/CD3/4/CD28+CD45RA-CCR7-
21	/Singlets/45/Lymphocytes/CD3/4/28-CD45RA+CCR7+
22	/Singlets/45/Lymphocytes/CD3/4/28-CD45RA+CCR7-
23	/Singlets/45/Lymphocytes/CD3/4/28-CD45RA-CCR7+
24	/Singlets/45/Lymphocytes/CD3/4/28-CD45RA-CCR7-
25	/Singlets/45/Lymphocytes/CD3/4/CD45RA+
26	/Singlets/45/Lymphocytes/CD3/4/278+
27	/Singlets/45/Lymphocytes/CD3/4/CCR7+
28	/Singlets/45/Lymphocytes/CD3/4/HLADR+
29	/Singlets/45/Lymphocytes/CD3/4/PD1+
30	/Singlets/45/Lymphocytes/CD3/4/CD45RA+ICOS+CCR7+
31	/Singlets/45/Lymphocytes/CD3/4/CD45RA+ICOS+CCR7-
32	/Singlets/45/Lymphocytes/CD3/4/CD45RA+ICOS-CCR7+
33	/Singlets/45/Lymphocytes/CD3/4/CD45RA+ICOS-CCR7-
34	/Singlets/45/Lymphocytes/CD3/4/CD45RA+CCR7+
35	/Singlets/45/Lymphocytes/CD3/4/CD45RA+CCR7+HLADR+
36	/Singlets/45/Lymphocytes/CD3/4/CD45RA+CCR7+HLADR-
37	/Singlets/45/Lymphocytes/CD3/4/CD45RA+CCR7+PD1+
38	/Singlets/45/Lymphocytes/CD3/4/CD45RA+CCR7+PD1-
39	/Singlets/45/Lymphocytes/CD3/4/CD45RA+CCR7-
40	/Singlets/45/Lymphocytes/CD3/4/CD45RA+CCR7-HLADR+
41	/Singlets/45/Lymphocytes/CD3/4/CD45RA+CCR7-HLADR-
42	/Singlets/45/Lymphocytes/CD3/4/CD45RA+CCR7-PD1+

43	/Singlets/45/Lymphocytes/CD3/4/CD45RA+CCR7-PD1-
44	/Singlets/45/Lymphocytes/CD3/4/CD45RA-ICOS+CCR7+
45	/Singlets/45/Lymphocytes/CD3/4/CD45RA-ICOS+CCR7-
46	/Singlets/45/Lymphocytes/CD3/4/CD45RA-ICOS-CCR7+
47	/Singlets/45/Lymphocytes/CD3/4/CD45RA-ICOS-CCR7-
48	/Singlets/45/Lymphocytes/CD3/4/CD45RA-CCR7+
49	/Singlets/45/Lymphocytes/CD3/4/CD45RA-CCR7+HLADR+
50	/Singlets/45/Lymphocytes/CD3/4/CD45RA-CCR7+HLADR-
51	/Singlets/45/Lymphocytes/CD3/4/CD45RA-CCR7+PD1+
52	/Singlets/45/Lymphocytes/CD3/4/CD45RA-CCR7+PD1-
53	/Singlets/45/Lymphocytes/CD3/4/CD45RA-CCR7-
54	/Singlets/45/Lymphocytes/CD3/4/CD45RA-CCR7-HLADR+
55	/Singlets/45/Lymphocytes/CD3/4/CD45RA-CCR7-HLADR-
56	/Singlets/45/Lymphocytes/CD3/4/CD45RA-CCR7-PD1+
57	/Singlets/45/Lymphocytes/CD3/4/CD45RA-CCR7-PD1-
58	/Singlets/45/Lymphocytes/CD3/4/CD45RA-
59	/Singlets/45/Lymphocytes/CD3/4/treg
60	/Singlets/45/Lymphocytes/CD3/8
61	/Singlets/45/Lymphocytes/CD3/8/CD25+
62	/Singlets/45/Lymphocytes/CD3/8/CD25+CD45RA+CCR7+
63	/Singlets/45/Lymphocytes/CD3/8/CD25+CD45RA+CCR7-
64	/Singlets/45/Lymphocytes/CD3/8/CD25+CD45RA-CCR7+
65	/Singlets/45/Lymphocytes/CD3/8/CD25+CD45RA-CCR7-
66	/Singlets/45/Lymphocytes/CD3/8/CD25-CD45RA+CCR7+
67	/Singlets/45/Lymphocytes/CD3/8/CD25-CD45RA+CCR7-
68	/Singlets/45/Lymphocytes/CD3/8/CD25-CD45RA-CCR7+
69	/Singlets/45/Lymphocytes/CD3/8/CD25-CD45RA-CCR7-
70	/Singlets/45/Lymphocytes/CD3/8/CD28+
71	/Singlets/45/Lymphocytes/CD3/8/CD28+CD45RA+CCR7+
72	/Singlets/45/Lymphocytes/CD3/8/CD28+CD45RA+CCR7-
73	/Singlets/45/Lymphocytes/CD3/8/CD28+CD45RA-CCR7+
74	/Singlets/45/Lymphocytes/CD3/8/CD28+CD45RA-CCR7-
75	/Singlets/45/Lymphocytes/CD3/8/28-CD45RA+CCR7+
76	/Singlets/45/Lymphocytes/CD3/8/28-CD45RA+CCR7-
77	/Singlets/45/Lymphocytes/CD3/8/28-CD45RA-CCR7+
78	/Singlets/45/Lymphocytes/CD3/8/28-CD45RA-CCR7-
79	/Singlets/45/Lymphocytes/CD3/8/CD45RA+
80	/Singlets/45/Lymphocytes/CD3/8/278+
81	/Singlets/45/Lymphocytes/CD3/8/CCR7+
82	/Singlets/45/Lymphocytes/CD3/8/HLADR+
83	/Singlets/45/Lymphocytes/CD3/8/PD1+
84	/Singlets/45/Lymphocytes/CD3/8/CD45RA+ICOS+CCR7+
85	/Singlets/45/Lymphocytes/CD3/8/CD45RA+ICOS+CCR7-
86	/Singlets/45/Lymphocytes/CD3/8/CD45RA+ICOS-CCR7+
87	/Singlets/45/Lymphocytes/CD3/8/CD45RA+ICOS-CCR7-
88	/Singlets/45/Lymphocytes/CD3/8/CD45RA+CCR7+

89	/Singlets/45/Lymphocytes/CD3/8/CD45RA+CCR7+HLADR+
90	/Singlets/45/Lymphocytes/CD3/8/CD45RA+CCR7+HLADR-
91	/Singlets/45/Lymphocytes/CD3/8/CD45RA+CCR7+PD1+
92	/Singlets/45/Lymphocytes/CD3/8/CD45RA+CCR7+PD1-
93	/Singlets/45/Lymphocytes/CD3/8/CD45RA+CCR7-
94	/Singlets/45/Lymphocytes/CD3/8/CD45RA+CCR7-HLADR+
95	/Singlets/45/Lymphocytes/CD3/8/CD45RA+CCR7-HLADR-
96	/Singlets/45/Lymphocytes/CD3/8/CD45RA+CCR7-PD1+
97	/Singlets/45/Lymphocytes/CD3/8/CD45RA+CCR7-PD1-
98	/Singlets/45/Lymphocytes/CD3/8/CD45RA-ICOS+CCR7+
99	/Singlets/45/Lymphocytes/CD3/8/CD45RA-ICOS+CCR7-
100	/Singlets/45/Lymphocytes/CD3/8/CD45RA-ICOS-CCR7+
101	/Singlets/45/Lymphocytes/CD3/8/CD45RA-ICOS-CCR7-
102	/Singlets/45/Lymphocytes/CD3/8/CD45RA-CCR7+
103	/Singlets/45/Lymphocytes/CD3/8/CD45RA-CCR7+HLADR+
104	/Singlets/45/Lymphocytes/CD3/8/CD45RA-CCR7+HLADR-
105	/Singlets/45/Lymphocytes/CD3/8/CD45RA-CCR7+PD1+
106	/Singlets/45/Lymphocytes/CD3/8/CD45RA-CCR7+PD1-
107	/Singlets/45/Lymphocytes/CD3/8/CD45RA-CCR7-
108	/Singlets/45/Lymphocytes/CD3/8/CD45RA-CCR7-HLADR+
109	/Singlets/45/Lymphocytes/CD3/8/CD45RA-CCR7-HLADR-
110	/Singlets/45/Lymphocytes/CD3/8/CD45RA-CCR7-PD1+
111	/Singlets/45/Lymphocytes/CD3/8/CD45RA-CCR7-PD1-
112	/Singlets/45/Lymphocytes/CD3/8/CD45RA-

This table records all nodes derived in the manual gating strategy applied to CITN-09 T cell panel.

Table S23: CITN-09 Myeloid Manual Gating Strategy

1	root
2	/Singlets
3	/Singlets/45+
4	/Singlets/45+/CD3-CD19-
5	/Singlets/45+/CD3-CD19-/CD20-
6	/Singlets/45+/CD3-CD19-/CD20-/CD56-
7	/Singlets/45+/CD3-CD19-/CD20-/CD56-/HLADR-
8	/Singlets/45+/CD3-CD19-/CD20-/CD56-/HLADR-/CD16-
9	/Singlets/45+/CD3-CD19-/CD20-/CD56-/HLADR-/CD16-/CD14+
10	/Singlets/45+/CD3-CD19-/CD20-/CD56-/HLADR-/CD16-/CD14+/Q1: CD33-, CD11B+
11	/Singlets/45+/CD3-CD19-/CD20-/CD56-/HLADR-/CD16-/CD14+/Q2: CD33+, CD11B+ (m-MDSC)
12	/Singlets/45+/CD3-CD19-/CD20-/CD56-/HLADR-/CD16-/CD14+/Q3: CD33+, CD11B-
13	/Singlets/45+/CD3-CD19-/CD20-/CD56-/HLADR-/CD16-/CD14+/Q4: CD33-, CD11B-
14	/Singlets/45+/CD3-CD19-/CD20-/CD56-/HLADR-/CD16-/CD14-CD15+
15	/Singlets/45+/CD3-CD19-/CD20-/CD56-/HLADR-/CD16-/CD14-CD15+/Q1: CD33-, CD11B+
16	/Singlets/45+/CD3-CD19-/CD20-/CD56-/HLADR-/CD16-/CD14-CD15+/Q2: CD33+, CD11B+ (PMN-MDSC)
17	/Singlets/45+/CD3-CD19-/CD20-/CD56-/HLADR-/CD16-/CD14-CD15+/Q3: CD33+, CD11B-
18	/Singlets/45+/CD3-CD19-/CD20-/CD56-/HLADR-/CD16-/CD14-CD15+/Q4: CD33-, CD11B-
19	/Singlets/45+/CD3-CD19-/CD20-/CD56-/HLADR-/CD16-/CD14-CD15-
20	/Singlets/45+/CD3-CD19-/CD20-/CD56-/HLADR-/CD16-/CD14-CD15-/Q1: CD33-, CD11B+
21	/Singlets/45+/CD3-CD19-/CD20-/CD56-/HLADR-/CD16-/CD14-CD15-/Q2: CD33+, CD11B+ (e-MDSC)
22	/Singlets/45+/CD3-CD19-/CD20-/CD56-/HLADR-/CD16-/CD14-CD15-/Q3: CD33+, CD11B-
23	/Singlets/45+/CD3-CD19-/CD20-/CD56-/HLADR-/CD16-/CD14-CD15-/Q4: CD33-, CD11B-
24	/Singlets/45+/CD14+
25	/Singlets/45+/CD14-CD15+
26	/Singlets/45+/CD14-CD15-

Table S23: This table records all nodes derived in the manual gating strategy applied to CITN-09 Myeloid panel.

Table S24: CITN-07 Phenotyping Manual Gating Strategy

1	root
2	/Beads
3	/Non-beads
4	/Non-beads/Singlets
5	/Non-beads/Singlets/45+
6	/Non-beads/Singlets/45+/14+
7	/Non-beads/Singlets/45+/14-
8	/Non-beads/Singlets/45+/14-/3-19-
9	/Non-beads/Singlets/45+/14-/3-19-/56-16-
10	/Non-beads/Singlets/45+/14-/3-19-/56-16-/Basophils
11	/Non-beads/Singlets/45+/14-/3-19-/56-16-/Basophils/HLA DR hi
12	/Non-beads/Singlets/45+/14-/3-19-/56-16-/Basophils/HLA DR med
13	/Non-beads/Singlets/45+/14-/3-19-/56-16-/Basophils/HLA DR neg
14	/Non-beads/Singlets/45+/14-/3-19-/56-16-/HLADR+
15	/Non-beads/Singlets/45+/14-/3-19-/56-16-/HLADR+/mDC
16	/Non-beads/Singlets/45+/14-/3-19-/56-16-/HLADR+/mDC/HLADRhi
17	/Non-beads/Singlets/45+/14-/3-19-/56-16-/HLADR+/mDC/HLADRmed
18	/Non-beads/Singlets/45+/14-/3-19-/56-16-/HLADR+/mDC/HLA DR hi
19	/Non-beads/Singlets/45+/14-/3-19-/56-16-/HLADR+/mDC/HLA DR med
20	/Non-beads/Singlets/45+/14-/3-19-/56-16-/HLADR+/mDC/HLA DR neg
21	/Non-beads/Singlets/45+/14-/3-19-/56-16-/HLADR+/pDC
22	/Non-beads/Singlets/45+/14-/3-19-/56-16-/HLADR+/pDC/HLA DR hi
23	/Non-beads/Singlets/45+/14-/3-19-/56-16-/HLADR+/pDC/HLA DR med
24	/Non-beads/Singlets/45+/14-/3-19-/56-16-/HLADR+/pDC/HLA DR neg
25	/Non-beads/Singlets/45+/Lymphocytes
26	/Non-beads/Singlets/45+/Lymphocytes/3+
27	/Non-beads/Singlets/45+/Lymphocytes/3+/4&8
28	/Non-beads/Singlets/45+/Lymphocytes/3+/4&8/56+
29	/Non-beads/Singlets/45+/Lymphocytes/3+/4&8/122+
30	/Non-beads/Singlets/45+/Lymphocytes/3+/4&8++
31	/Non-beads/Singlets/45+/Lymphocytes/3+/4+
32	/Non-beads/Singlets/45+/Lymphocytes/3+/4+/HLADR+
33	/Non-beads/Singlets/45+/Lymphocytes/3+/8+
34	/Non-beads/Singlets/45+/Lymphocytes/3+/8+/HLADR+

35	/Non-beads/Singlets/45+/Lymphocytes/3-19-
36	/Non-beads/Singlets/45+/Lymphocytes/3-19-/16+56-
37	/Non-beads/Singlets/45+/Lymphocytes/3-19-/56+
38	/Non-beads/Singlets/45+/Lymphocytes/3-19-/56+/122+
39	/Non-beads/Singlets/45+/Lymphocytes/3-19-/56+16-
40	/Non-beads/Singlets/45+/Lymphocytes/3-19-/56-16-
41	/Non-beads/Singlets/45+/Lymphocytes/3-19-/56-16-/122+
42	/Non-beads/Singlets/45+/Lymphocytes/3-19-/56-16-/HLA DR hi
43	/Non-beads/Singlets/45+/Lymphocytes/3-19-/56-16-/HLA DR med
44	/Non-beads/Singlets/45+/Lymphocytes/3-19-/56-16-/HLA DR neg
45	/Non-beads/Singlets/45+/Lymphocytes/3-19-/56B
46	/Non-beads/Singlets/45+/Lymphocytes/3-19-/56B/HLA DR hi
47	/Non-beads/Singlets/45+/Lymphocytes/3-19-/56B/HLA DR med
48	/Non-beads/Singlets/45+/Lymphocytes/3-19-/56B/HLA DR neg
49	/Non-beads/Singlets/45+/Lymphocytes/3-19-/56B16-
50	/Non-beads/Singlets/45+/Lymphocytes/3-19-/56D
51	/Non-beads/Singlets/45+/Lymphocytes/3-19-/56D/HLA DR hi
52	/Non-beads/Singlets/45+/Lymphocytes/3-19-/56D/HLA DR med
53	/Non-beads/Singlets/45+/Lymphocytes/3-19-/56D/HLA DR neg
54	/Non-beads/Singlets/45+/Lymphocytes/19+
55	/Non-beads/Singlets/45+/Lymphocytes/19+/B CELLS
56	/Non-beads/Singlets/45+/Lymphocytes/19+/B CELLS/HLA DR hi
57	/Non-beads/Singlets/45+/Lymphocytes/19+/B CELLS/HLA DR med
58	/Non-beads/Singlets/45+/Lymphocytes/19+/B CELLS/HLA DR neg

This table records all nodes derived in the manual gating strategy applied to CITN-07 phenotyping panel.
